# Mutant p53^R211*^ ameliorates inflammatory arthritis in AIA rats via inhibition of TBK1-IRF3 innate immune response

**DOI:** 10.1007/s00011-023-01809-w

**Published:** 2023-11-08

**Authors:** Yaling Zeng, Jerome P. L. Ng, Linna Wang, Xiongfei Xu, Betty Yuen Kwan Law, Guobing Chen, Hang Hong Lo, Lijun Yang, Jiujie Yang, Lei Zhang, Liqun Qu, Xiaoyun Yun, Jing Zhong, Ruihong Chen, Dingqi Zhang, Yuping Wang, Weidan Luo, Congling Qiu, Baixiong Huang, Wenfeng liu, Liang Liu, Vincent Kam Wai Wong

**Affiliations:** 1 Dr. Neher’s Biophysics Laboratory for Innovative Drug Discovery, State Key Laboratory of Quality Research in Chinese Medicine, Macau University of Science and Technology, Macau, 999078 China; 2 Department of Microbiology and Immunology, Institute of Geriatric Immunology, School of Medicine, Jinan University, Guangzhou, 510630 China; 3 School of Biotechnology and Health Sciences, Wuyi University, Jiangmen, 529020 China

**Keywords:** p53 mutant, Rheumatoid arthritis, TBK1, IRF3, STING

## Abstract

**Background:**

Rheumatoid arthritis (RA) is an autoimmune inflammation disease characterized by imbalance of immune homeostasis. p53 mutants are commonly described as the guardian of cancer cells by conferring them drug-resistance and immune evasion. Importantly, p53 mutations have also been identified in RA patients, and this prompts the investigation of its role in RA pathogenesis.

**Methods:**

The cytotoxicity of disease-modifying anti-rheumatic drugs (DMARDs) against p53 ^wild-type (WT)/mutant^-transfected RA fibroblast-like synoviocytes (RAFLSs) was evaluated by MTT assay. Adeno-associated virus (AAV) was employed to establish p53 ^WT/R211*^ adjuvant-induced arthritis (AIA) rat model. The arthritic condition of rats was assessed by various parameters such as micro-CT analysis. Knee joint samples were isolated for total RNA sequencing analysis. The expressions of cytokines and immune-related genes were examined by qPCR, ELISA assay and immunofluorescence. The mechanistic pathway was determined by immunoprecipitation and Western blotting in vitro and in vivo.

**Results:**

Among p53 mutants, p53^R213*^ exhibited remarkable DMARD-resistance in RAFLSs. However, AAV-induced p53^R211*^ overexpression ameliorated inflammatory arthritis in AIA rats without Methotrexate (MTX)-resistance, and our results discovered the immunomodulatory effect of p53^R211*^ via suppression of T-cell activation and T helper 17 cell (Th17) infiltration in rat joint, and finally downregulated expressions of pro-inflammatory cytokines. Total RNA sequencing analysis identified the correlation of p53^R211*^ with immune-related pathways. Further mechanistic studies revealed that p53^R213*/R211*^ instead of wild-type p53 interacted with TANK-binding kinase 1 (TBK1) and suppressed the innate immune TBK1–Interferon regulatory factor 3 (IRF3)–Stimulator of interferon genes (STING) cascade.

**Conclusions:**

This study unravels the role of p53^R213*^ mutant in RA pathogenesis, and identifies TBK1 as a potential anti-inflammatory target.

**Supplementary Information:**

The online version contains supplementary material available at 10.1007/s00011-023-01809-w.

## Introduction

Rheumatoid arthritis (RA) is a chronic autoimmune disease with obvious clinical symptom of persistent synovitis and joint damage [1]. Dysregulation of multiple immune cells recognize autoantigens, generate autoantibodies and inflammatory cytokines which in turn promote autoimmune responses [1, 2]. Activated immune cells infiltrate in joint tissue, facilitate the proliferation of synovial fibroblast and secretion of metalloprotease, finally result in progressive destruction of articular cartilage and subchondral bone [2]. During the early stage of RA onset, the activated autoimmune response starts with the T-cell recognition of self-antigens expressed on antigen-presenting cells, and subsequently stimulates CD4^+^ T cells to differentiate into T helper (Th) cells, arising unusually high Th17 population [2]. The imbalance of regulatory T (Treg)/Th17 cell ratio then promotes the interaction of immune cells with synovial fibroblasts, and eventually aggravates joint inflammation [2]. Moreover, Th17 cell also plays a crucial role in the pathogenesis of RA by producing IL-17 and other pro-inflammatory cytokines [3]. Along with RA progression, it finally causes irreversible joint disability and other organ damage, such as heart, lungs, kidney, and even nervous and vascular systems [1, 4]. Furthermore, the long-term use of disease-modifying anti-rheumatic drugs (DMARDs) such as Methotrexate (MTX), Leflunomide (LEF) and Cyclosporine A (CSA) for RA promotes the development of drug resistance, thus giving rise to refractory RA [5]. The occurrence of refractory RA finally affects the quality of life in RA patients [6], and therefore, it is of utmost importance to develop new treatment strategies based on understanding of RA pathogenesis.

p53 is known as a key tumor suppressor gene that regulates cell cycle arrest, DNA repair, apoptosis, etc. [7]. p53 also plays a critical role in inflammatory and immune responses, responsible for expressions of multiple cytokines and matrix metalloproteinases (MMPs) [8, 9]. Besides, p53 has been reported to mediate immune-related genes, such as CC-chemokine ligand 2 (CCL2), interferon regulatory factor 5 (IRF5), IRF9 and Toll-like receptor 3 (TLR3) [10]. Given the importance of p53 in homeostatic regulation of immune responses, malfunction of p53, due to its deficient expression and mutation, exerts detrimental effect in the pathogenesis of cancer and autoimmune diseases [11, 12]. Notably, clinical studies revealed that variable p53 mutation rate was found in cancer cells as well as synovial fibroblast [13, 14]. These p53 mutations usually occurred at residues 94–312 located within central DNA binding domain (DBD), which reduced the binding affinity towards target gene promoters [15, 16]. As a consequence, mutant p53 inactivates tumor suppressor function, promotes proliferation, invasiveness, immune evasion and development of multidrug resistance (MDR) [11, 17, 18]. MDR1 was the first ATP-binding cassette (ABC) transporter associated with multidrug-resistance (MDR), which can be specifically activated by mutant p53 via ETS-binding site in MDR1 promotor region, thereby initiating the transcription of MDR1. Accordingly, drug resistance induced by mutant p53 mainly through ABC transporter-mediated efflux of drugs. Moreover, mutations of p53 have been reported to prevent drug-induced cell apoptosis, promote cells proliferation, activate DNA repair and inhibit autophagy to enhance microenvironmental resistance [19, 20]. Apparently, these drug resistance and apoptosis-resistance mediated by mutant p53 might eventually drive the disease progression to drug resistant and refractory stage [21, 22]. Although intensive studies have been carried out in cancer, the mechanism underlying p53 mutant-mediated MDR in RA is yet to elucidate.

Among various p53 mutations, R213* was identified as the most common of p53 nonsense mutation hotspot in cancer. For example, p53^R213*^ mutant upregulated IL-6 expression and downregulated Bax promoter activity in HS68 cells [11, 20, 23]. p53^R213*^ also resulted in cell cycle arrest via downregulation of p21 expression in H1299 [24]. Moreover, p53^R213*^ promoted the tumor growth and metastasis in Melanomas [25]. In contrast to the aforementioned studies in cancers, the role of p53^R213*^ in the pathogenesis of RA remains unexplored. In accordance with our preliminary in vitro results, the most promising p53^R211*^ (human p53^R213*^) mutant was selected for further examining its effect in adjuvant-induced arthritis (AIA) rats. Surprisingly, we unraveled that this mutant showed immunomodulatory effect in AIA rats, and the underlying mechanism of TBK1 inhibition was reported.

## Materials and methods

### Cell transfection with p53 wide type and mutants

Plasmids of human p53^WT^ (GeneBank: NM_000546.6) and various mutations were constructed in our laboratory. RAFLS were transfected with p53^WT^ and p53 mutants by using Lipofectamine® 3000 (Invitrogen). 5 × 10^5^ RAFLS cell were seeded per well in a 6-well plate for overnight culture. 125 µl of Opti-MEMTM I medium, 4 µl of Lipofectamine 3000 reagent, 4 µl of P3000TM reagent and 2 μg DNA plasmid were added to each well, then changed the medium after 8 h. Cells were harvested after transfection for 48 h.

### Cytotoxicity assays

All tested compounds were dissolved in DMSO to a final concentration of 100 mmol/L (HCQ, CSA), 500 mmol/L (FK506) and 1000 mmol/L (LEF)  respectively. 6 × 10^4^ RAFLSs were seeded per well in a 96-well plate. After overnight culture, the cells were treated with compounds for 72 h. 10 µl of MTT was added to each well. After incubation at 37 ℃ for 4 h, 100 µl of solubilization buffer (10% SDS in 0.01 mol/L HCI) was added and incubated for overnight. The OD values were measured at 575 nm wavelength (A_575_). The percentage of cell viability was calculated using the following formula: cell survival (%) = A_treated_/A_control_ × 100. Data were obtained from three independent experiments.

### RNA extraction and cDNA reverse transcription

Total RNA of frozen rat joint tissues was extracted using TRIzol (Invitrogen, USA). After quickly frozen in liquid nitrogen, approximately 40–60 mg crushed tissues were added in 1 ml of TRIzol. 200 µl chloroform was added and shaked vigorously, then the mixture was centrifuged at 12,000 rpm for 10 min at 4 °C. Supernatant and isopropanol was added in a ratio of 1:1.2 (supernatant:isopropanol) to a new tube, then centrifuged at 12,000 rpm for 10 min at 4 °C. After removal of supernatant, the precipitate was washed with 75% cold ethanol, and finally 50 µl of RNase-free water was added to dissolve the precipitate. UV spectrophotometry (NanoDrop Technologies, USA) was used to measure the quality and concentration of RNA. 1 μg of total RNA was reverse transcribed to corresponding cDNA using Maxima™ H Minus cDNA Synthesis Master Mix (Thermo, USA).

### Real-time quantitative PCR

A total of 10 µl of PCR mixture consisted of 1 µl of cDNA, 0.2 µl of forward and reverse primers  respectively, 0.2 µl of Dye, 5 µl of SYBR Master Mix (Roche Diagnostics, USA), and 3.4 µl of ddH_2_O. Quantification of gene expression was determined by ViiA 7 Real-Time PCR System (Applied Biosystems). Data were normalized to β-actin and analysed using the 2^−ΔΔCT^ method.

### Western blot

The cells were lysed with RIPA lysis buffer (1 × Protease inhibitor cocktail from Roche; 100 mM of PMSF, 150 mM of NaCl, 100 mM of DTT; 50 mM of Tris–HCl, pH 7.5; 0.5 Mm of EDTA) for 20 min. The soluble fractions were collected after centrifugation at 12,000 rpm for 15 min at 4 °C. Protein concentrations were measured using Bio-Rad protein assay. 30 µg of protein was separated by 10% SDS-PAGE then transferred onto PVDF membranes (Bio-Rad, USA) and blocked with 5% non-fat-dry milk for 1 h. These membranes were stained with primary antibodies overnight at 4 °C. After washing the membranes using TBST thrice, the membranes were incubated with anti-rabbit or anti-mouse secondary antibodies (1:2000; Santa Cruz Biotechnology) for 2 h at room temperature. ELC assay by FluorChem R (ProteinSimple, America) was used to visualize the hybridized bands under the Amersham Imager 600 (GE) Imaging System. Band densities quantification was analyzed by Image J software.

### Establishment of adjuvant-induced arthritis (AIA) in SD rat and treatment

Male Sprague–Dawley (SD) rats weighing 80–100 g were purchased from SPF Biotechnology Co., Ltd (Beijing, China). Rats were divided into seven groups (*n* = 8–10) as following: (1) Healthy control (*n* = 8), (2) AIA + AAV-EGFP (Vehicle control, *n* = 8), (3) AIA + Methotrexate (MTX 7.6 mg/kg) (*n* = 8), (4) AIA + AAV-p53^WT^ (1 × 10^11^ PFU for each joint) (*n* = 10), (5) AIA + AAV-p53^WT^ + MTX (*n* = 8), (6) AIA + AAV-p53^R211*^ (*n* = 10), (7) AIA + AAV-p53^R211*^ + MTX (*n* = 8). pAAV-CMV-p53-3 × FLAG-EF1-mNeonGreen-WPRE (Ad-p53^WT^), pAAV-CMV-p53^R211*^-3 × FLAG-EF1-mNeonGreen-WPRE and vehicle control Adeno-associated virus (AAV) were constructed and packaged by OBiO technology (ShangHai, China). One week before AIA induction, 30 µl AAV-EGFP or AAV-P53^R211*^ were injected into each knee-joint of each rat. For establishment of AIA model, *mycobacterium tuberculosis (M. tuberculosis DES. H37 RA, DIFCO, USA)* was emulsified in mineral oil (Sigma) to yield 2.5 mg/ml of *M. tuberculosis*. Rats were injected with 0.1 ml of this emulsion subcutaneously at the base of the tail on Day 0. Rats received MTX treatment (7.6 mg/kg) by gavage once a week. Arthritic scores and hind paw volume were evaluated and recorded every 3 days. On Day 28, these rats were sacrificed, blood, organs, and joint tissue were harvested for biochemical assays and micro-CT analysis.

### Assessment of joint swelling and arthritic scores

The volume of hind paw and arthritic scores were evaluated every 3 days. The investigator was blinded to the group allocation when measuring the joint swelling. The volume of hind paws was measured using Plethysmometer (Ugo Basile, Italy or Kent Scientific Corp, Connecticut USA). Clinical scores were obtained for each paw according to the following standards the severity of arthritis was objectively inspected on four paws and was scored on a scale of 0–4 for each paw according to the arthritis index: score 0 means there was no swelling and erythema evidence on the joint, including the small joints of the front foot and phalangeal joint, large joints including wrist and ankle; score 1 means there was mild swelling and erythema on ankle; score 2 means mild swelling and erythema extended to small joint; score 3 means there was serious swelling and erythema on large joint; score 4 means serious swelling and erythema encompassing on small and large joint.

### Micro-CT analysis

At the end of treatment period, the right hind paw of sacrificed rats was amputated and fixed in 4% PFA, then scanned using in vivo micro-CT scanner (SkyScan 1176, Bruker, Belgium). The following scanning parameters were used to obtain high-quality images of the rat joint: 35 µm resolution, 85 kV, 385 µA, 65 ms exposure time, 0.7, rotation step in 360°, and a 1 mm Al filter. The images were reconstructed using NRecon software (Bruker-micro-CT, Belgium). Bone density was analyzed by CT An software. Micro-CT score was calculated from five disease-related indexes of the micro-CT analysis for calcaneus, including bone mineral density, bone volume fraction, cortical mineral density, trabecular number, and total porosity using the following formula: (Acquired value − minimum value)/(maximum value − minimum value) or 1-(Acquired value − minimum value)/(maximum value − minimum value). The final micro-CT score is equally averaged from the above five aspects of bones (Micro-CT score: 0–0.2, mutilating; 0.4–0.6, moderate; 0.8–1, normal). According to the severity of bone erosion, the bone condition of rats was evaluated with radiological score.

### Hematoxylin and eosin (H&E) and immunofluorescent (IF) staining

Joint tissues were fixed in 4% paraformaldehyde for 24 h and subjected to dehydration. The tissues were embedded in paraffin for microtome sectioning and H&E or IF. Joint sections (6 μm thickness) were dehydrated, deparaffinized and rehydrated. For H&E staining, joint tissues were subjected to treatment with hematoxylin and eosin. For IF, joint tissues were incubated with primary antibody at 4 ℃ overnight. The secondary antibody (anti-rabbit FITC, anti-mouse Cy5, CST) was incubated at room temperature in the absence of light for 2 h. The coverslips were mounted with FluorSave™ Reagent (Millipore, USA). H&E images were captured by light microscopy (Leica DM2500, Germany). Fluorescent images were captured by API Delta Vision Live-Cell Imaging System.

### Flow cytometric analysis of lymphocytes

Peripheral blood mononuclear cells (PBMCs) were isolated from peripheral blood of rats using a standard Ficoll density-gradient centrifugation kit (GE Healthcare). The collected spleen was washed with PBS and grinded to collect lymphocytes. T cells were stained with the following antibodies: anti-rat CD45 (APC/Cyanine7); anti-rat CD3 (FITC); anti-rat CD4 (PerCP/Cyanine5.5); anti-rat CD8a (APC). For Treg cell analysis, cells were stained with anti-CD3, anti-CD4, anti-CD8, and anti-CD45 antibodies, then were washed, fixed, and permeabilized prior to staining with anti- Foxp3 (PE) antibody. For Th17 cell analysis, after stained with surface markers (anti-CD3, anti-CD4, anti-CD8, and anti-CD45), cells were fixed and permeabilized prior to staining withanti-IL-17A antibody (PE-Cyanine 7). All samples were measured by FACSAria III flow cytometer (BD Bioscience) and analyzed using the FlowJo v10 software (TreeStar, Inc.).

### LEGENDplex assay kit

Serum was collected from rat blood by centrifuging it at 3500 rpm for 10 min. The concentration of IL-10, IFN-γ, KC, TNF-α, IL-18, IL-12p70, IL-1β, IL-17A, IL-1α, GM-CSF, IL-33 and IL-6 in rat serum were determined using LEGENDplex (Biolegend, USA) kit according to the manufacturer’s instruction. The samples were analyzed by FACSAria III flow cytometer (BD Bioscience) and LEGENDplex ™ Data Analysis Version 8.0 software.

### Immunoprecipitation

The antibody (5 µg) was vortex-mixed with 50 µl immune-magnetic bead (Thermo, USA) and incubated at 4 ℃ overnight. The extracted protein was then added to the mixture and incubated at 4 ℃ for another overnight. Subsequently, the bead mixture was transferred to a clean tube and placed in a magnetic separation rack. The supernatant was discarded, and the residue was washed with washing buffer by using magnetic separation. The Dynabeads-Ab-Ag complex was re-suspended in 60 µl of elution buffer. 20 µl of 5 × loading buffer was added, and the resulting mixture was incubated for 10 min at 100 °C. The supernatant was separated by magnetic separation and stored at − 80 ℃.

### ELISA

Cytokines expressions in rat serum (50 µl for each sample) were detected by using specific Quantikine enzyme-linked immunosorbent assay (ELISA) kit (MEIMIAN, China) according to manufacturer’s instruction. The absorbance was measured at 450 nm using SpectraMax Paradigm (Molecular Devices, USA).

### Nuclear and cytoplasmic extraction

The transfected cells were harvested and transferred to a clean tube, followed by centrifugation at 500*g* for 5 min. Ice-cold CER I was added to the cell pellet and incubated on ice for 10 min. The cell extract was then vortex-mixed for 15 s, followed by centrifugation at maximum speed for 5 min. The supernatant (cytoplasmic protein) was transferred to a clean ice-cold tube. After that, ice-cold NER was mixed with insoluble residue in the tube, and the tube was placed on ice with vortex-mixing for 15 s at an interval of 10 min for 40 min in total. Subsequently, the tube was centrifuged at maximum speed for 10 min and transferred the supernatant to a new ice-cold tube (nuclear protein). Both extracts were stored at − 80 ℃.

### Total RNA sequencing and analysis

Collected rat knee joint were sent to LC-BIO Technologies Co., LTD (Hangzhou, China) for RNA extraction, library preparation and sequencing on Illumina NovaSeq 6000 plateform, following their standard procedure. DEGs analyses were performed by DESeq2 1.30.1 [26], after interpretation of DESeq2, DEGs were screened from the data with *p* value <  = 0.05 and fold change >  = 2 or foldchange <  = -2. The distribution of data was presented in a volcano plot which builds by the plot function in R 4.2.1 [27]. KEGG enrichment analysis was performed by R package ClusterProfiler v3.18.1 [28]. The p-value Cut-off for enrichGO was set to 0.05. DEGs data were further input into the KEGG gene tools [29] and converted into KEGG Orthology ID [30], the converted IDs combined with corresponding gene symbols were entered into the KEGG Mapper–Reconstruct tools [31] to identify the respective classifications, to visualize the classification analysis results, ggplot2 package (version 3.3.6) [32] were applied in R 4.2.1 [27] for the construction of KEGG Classification chart. STRING analysis was performed using Multiple Protein modules. After the network generated by STRING, while all other parameters were statically unchanged, the active interaction sources were set as only the Experiments and Database (which was denoted as Curated Database at the retrieve version of STRING).

### Statistical analysis

T test was used to analyze two groups. Over 3 or more groups were analyzed by one-way analysis of variance. All results are expressed as the means ± SEMs. GraphPad Prism 7.0 software was used to evaluate the statistical data. *p* < 0.05 was considered significant.

### Role of funding source

Funders of this study did not have any role in study design, data collection, data analysis, data interpretation, or writing of the report.

## Results

### Mutations of p53 contribute the drug-resistant effect of DMARDs in RAFLS

**Fig. 1:**
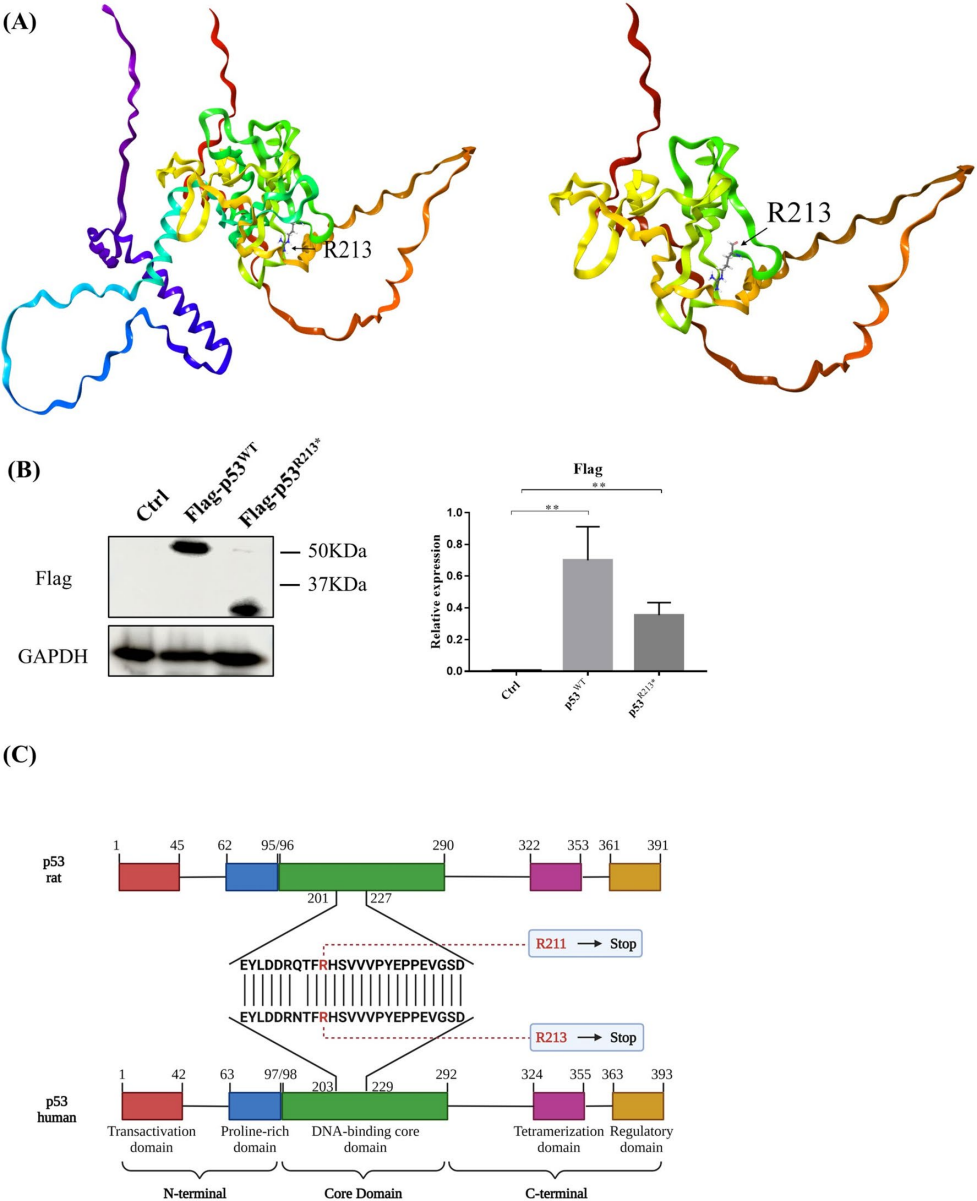
The general features of p53^WT^ and p53^R213*^. **A** The discrepancy between p53^WT^ and p53^R213*^ structural proteins. The structure was downloaded from the AlphaFold Protein Structure Database and modified as WT (which is the original structure) and R213* structure and further prepared by Protein Preparation wizard [37, 38]. **B** Expression level of Flag in RAFLS transfected with p53^WT^ or mutant p53^R213*^ plasmid. Cell lysates were collected and analyzed by Western blotting using antibodies against Flag. The bar chart shows the quantitation of the target protein expression with respect to actin expression using ImageJ software. The data are expressed as the mean ± SEM. One-way ANOVA; **p < 0.01 compared with control. **C** The amino acid mutation spectrum of p53^R213*^ in human and that of the corresponding p53^R211*^ in rat. Structures of p53 wild-type gene in human and rat are shown with numbered domain, and the color represents different regions of DNA domains. Human p53 mutant R213 or its corresponding rat mutant R211 is located at the DNA-binding core domain (green) (color figure online)

Previous studies demonstrated that mutations of tumor suppressor p53 were correlated with apoptosis-resistance and cell proliferation in various cancer and fibroblast-like synoviocytes derived from RA patients [33, 34]. We therefore selected and cloned the 123 different p53 mutants based on the reported p53 mutation sites identified in RA patients and investigated their drug-resistant effect in RA fibroblast-like synoviocytes (RAFLS). Our preliminary results revealed that several p53 mutations facilitated apoptosis-resistance and cell proliferation, as well as causing Methotrexate (MTX)-resistance in RAFLS. Among the selected p53 mutants, a nonsense mutant p53^R213*^ demonstrated significant anti-apoptotic activity and MTX-resistance in vitro [35]. Whilst p53 missense mutants, such as p53^R248Q^, have been reported for the loss of tumor suppression function and promotion of tumorigenesis [36], nonsense mutations are not frequently studied. In current study, we visualized the differences in protein conformations of p53 wild type (WT) and R213* mutant, the structures were predicted and constructed by AlphaFold (Fig. 1A) [37, 38]. Apparently, the nonsense mutant p53^R213*^ contains stop codon at site 213 of the p53 amino acid sequence, thereby shortening of amino acid and reducing the expression size of FLAG-p53^R213*^ in comparison with FLAG-p53^WT^ in RAFLS (Fig. 1B).

To further verify the drug-resistant effect induced by p53 mutants, disease-modifying anti-rheumatic drugs (DMARDs) such as Leflunomide (LEF), Cyclosporine A (CSA), Hydroxychloroquine (HCQ) and Tacrolimus (FK506) were used to examine their cytotoxicity against various mutants or wide type p53 transfected RAFLS. As shown in Supplementary Table S1, representative p53 mutants including p53^R213*^, p53^N239S^, p53^R248Q^, p53^R248W^ and p53^R342Q^ showed significant resistant factor (RF) ranging from 1.85 to 5.11 in CSA-, HCQ- or FK506-treated RAFLS. Moreover, these five p53 mutants showed high IC_50_ values for LEF. Interestingly, overexpression of p53^R213*^ induced drug-resistant effect to five DMARDs drugs and exhibited a strong anti-apoptotic effect in RAFLS. These findings prompted us to further investigate the drug resistant effect of p53^R213*^ in vivo, and therefore, adenovirus-mediated overexpression of rat p53^R211*^ (AAV-p53^R211*^) was employed to develop AIA rat model with overexpression of p53^R211*^. Notably, human p53 codons R213* corresponds to rat p53 codons R211* based on the amino acid sequence homology alignment (Fig. 1C).

### Intra-articular injection of AAV-p53^R211*^ ameliorated the arthritic conditions in AIA rats

To explore the effect of p53 mutant R211* in AIA rats, overexpression of p53^R211*^ mutant in synovium of AIA rat model was established by using the adeno-associated virus (AAV) as a gene delivery tool. AAV is one of the safest gene delivery strategies due to its long-term transgene expression, low immunogenicity and less pathogenicity in animals [39, 40]. Incorporation of target gene by serotype AAV like AAV2/5 in animal model has been well demonstrated in arthritis studies [41].

**Fig. 2:**
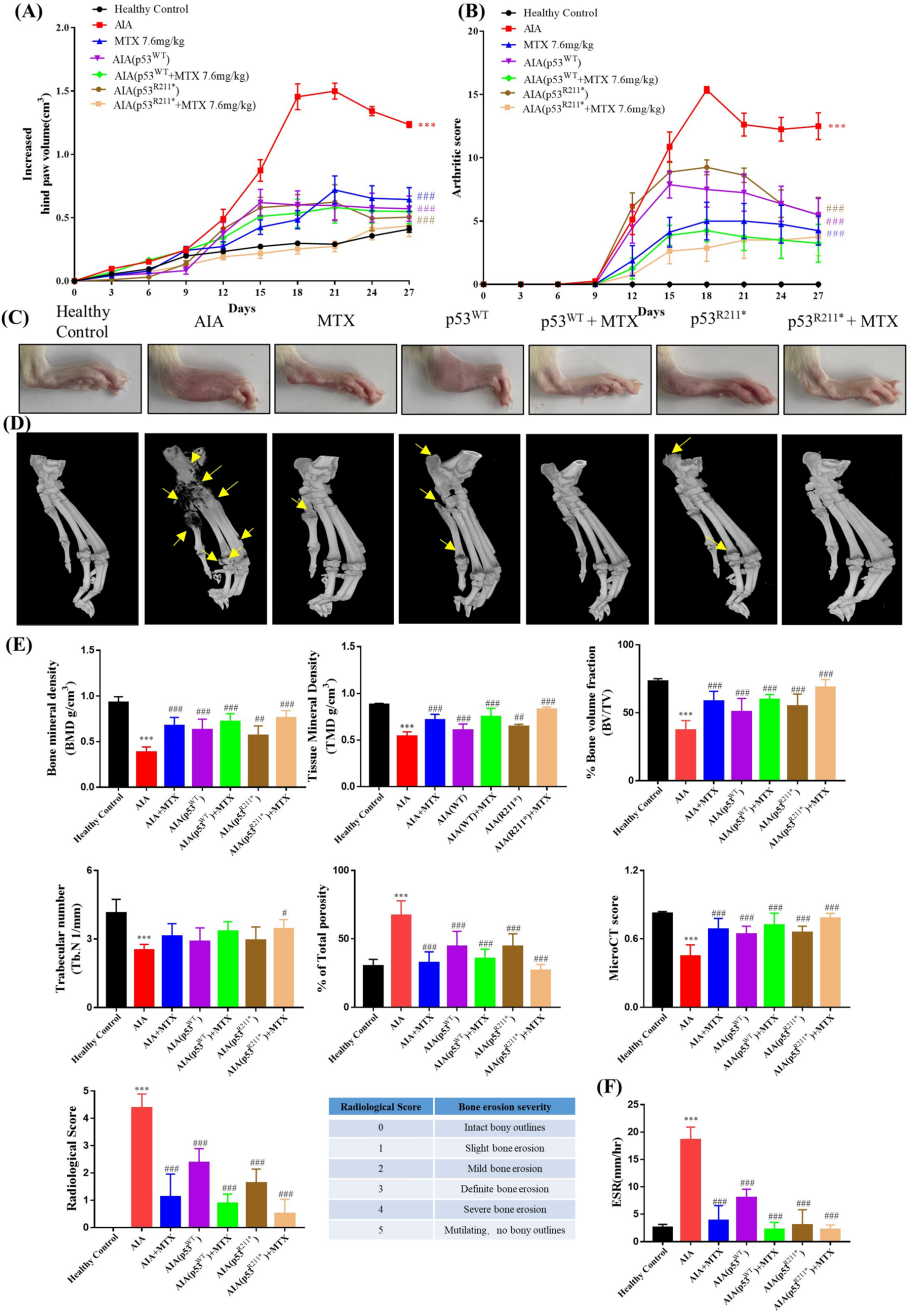
The suppression effect of adeno-associated virus (AAV)-p53^R211*^ in adjuvant-induced arthritis (AIA) rat model by single intra-articular knee injection. **A**, **B** The hind paw swelling and arthritic scores of AAV-p53^R211*^-injected AIA rat model. The AIA rats were either treated with the positive control drug, MTX (7.6 mg/kg), or articular knee injected with PBS, AAV-EGFP, AAV-p53^WT^ and AAV-p53^R211*^ (1 × 10^11^ PFU) with or without the co-treatment of MTX for 27 days. Hind paw volumes and arthritic scores were determined every 3 days. The data are expressed as mean ± SEM (*n* = 6–8). **C** Representative images of hind paw swelling on day 27 in AIA rats. **D** Representative micro-CT images of AIA rat hind paws (day 27) were reconstructed by using Inveon Research Workplace. The region of bone erosion was indicated by yellow arrows. **E** The micro-CT scores demonstrating the bone destruction condition of AIA rats were obtained from five disease-related indices: bone mineral density (BMD), tissue mineral density (TMD), % bone volume fraction (BV/TV), trabecular number (Tb. N), % of total porosity. Radiological scores were obtained by evaluating the bone erosion severity of rats with reference to the scoring table. The values are expressed as mean ± SEM (*n* = 6–8). **F** Erythrocyte sedimentation rate (ESR) in anticoagulated blood of all rat groups. WT: wild type, MTX: methotrexate. Two-way ANOVA; ^###^*p* < 0.001, ^##^*p* < 0.01, ^#^*p* < 0.05 compared with AIA control group; ***p < 0.001 compared with healthy controls (color figure online)

In this study, the inflammatory effect of p53^R211*^ mutant was evaluated for the arthritic conditions in adjuvant-induced arthritis (AIA) rats in terms of various parameters. Methotrexate (MTX), a frontline drug for clinical treatment of arthritis, was used as a positive control. As shown in Fig. 2A and 2B, the drastic increases in the arthritis score and hind paw volume indicated the onset of arthritis in SD rat model after day 9. Since loss of p53 was demonstrated to exacerbate collagen-induced arthritis and dysregulated the population of Th17 and Treg in mice [42]. In comparison with AIA control group, the AAV-p53^WT^ injection did show minimum improvement in the arthritic conditions in AIA rats. Notably, AAV-p53^R211*^ injection relieved the arthritis symptoms in AIA rats similar to the MTX treatment as reflected by the decreases in arthritis score and hind paw volume. The representative images of hind paw swelling also well illustrated the anti-arthritic effect of p53^R211*^ mutant (Fig. 2C).

Moreover, micro-CT analysis was further performed to assess the bone destruction effect of p53^R211*^ mutant in AIA rats. As shown in Fig. 2D, severe erosion and destruction of bone and cartilage were observed in AIA model rats. In line with the previous trend, AAV-p53^R211*^-injected group significantly ameliorated the arthritic conditions and improved the bone destruction. Micro-CT results were further quantified from numerous metrics including bone mineral density (BMD) (*p* = 0.0008), tissue mineral density (TMD) (*p* < 0.0001), % bone volume fraction (*p* = 0.0005), trabecular number (*p* = 0.0854) and % total porosity (*p* = 0.0005) (Fig. 2E). The mean micro-CT score improved remarkably from 0.44 to 0.65, whereas the radiological score was largely reduced from 4.37 to 1.62, suggesting the ameliorative effect of p53^R211*^ mutant in AIA rats. Besides, the erythrocyte sedimentation rate (ESR) was also significantly downregulated in the anticoagulated blood of AAV-p53^R211*^-injected group compared with AIA control (*p* < 0.0001) (Fig. 2F). Apparently, no adverse effect was observed in AAV-p53^WT/R211*^-injected groups upon co-treatment with MTX, implying that p53 wild-type (WT) or its mutant of R211* did not affect the effectiveness of MTX treatment. These results thus unraveled that p53^R211*^ mutant repressed arthritis symptoms in AIA rats.

### p53^R211*^ mutant suppressed T-cell activation and reduced Th17 cell populations in AIA rats

Systemic immune abnormality is a key factor in the occurrence and development of RA [43]. Dysregulation of immune system in RA is highly associated with excessive activation of T lymphocytes [44]. Cluster of differentiation 4 (CD4^+^) T cell is known to mediate cellular immunity and assist B cells to coordinate humoral immunity, whereas the increase in the CD4^+^/CD8^+^ ratio is frequently observed in RA patients, and it is therefore regarded as an important diagnostic measure of immune system functioning [44]. Importantly, the immune homeostasis relies on the balance of pro-inflammatory T helper 17 cell (Th17) and immunosuppressive regulatory T cell (Treg), which are differentiated from CD4^+^ cells [45].

**Fig. 3:**
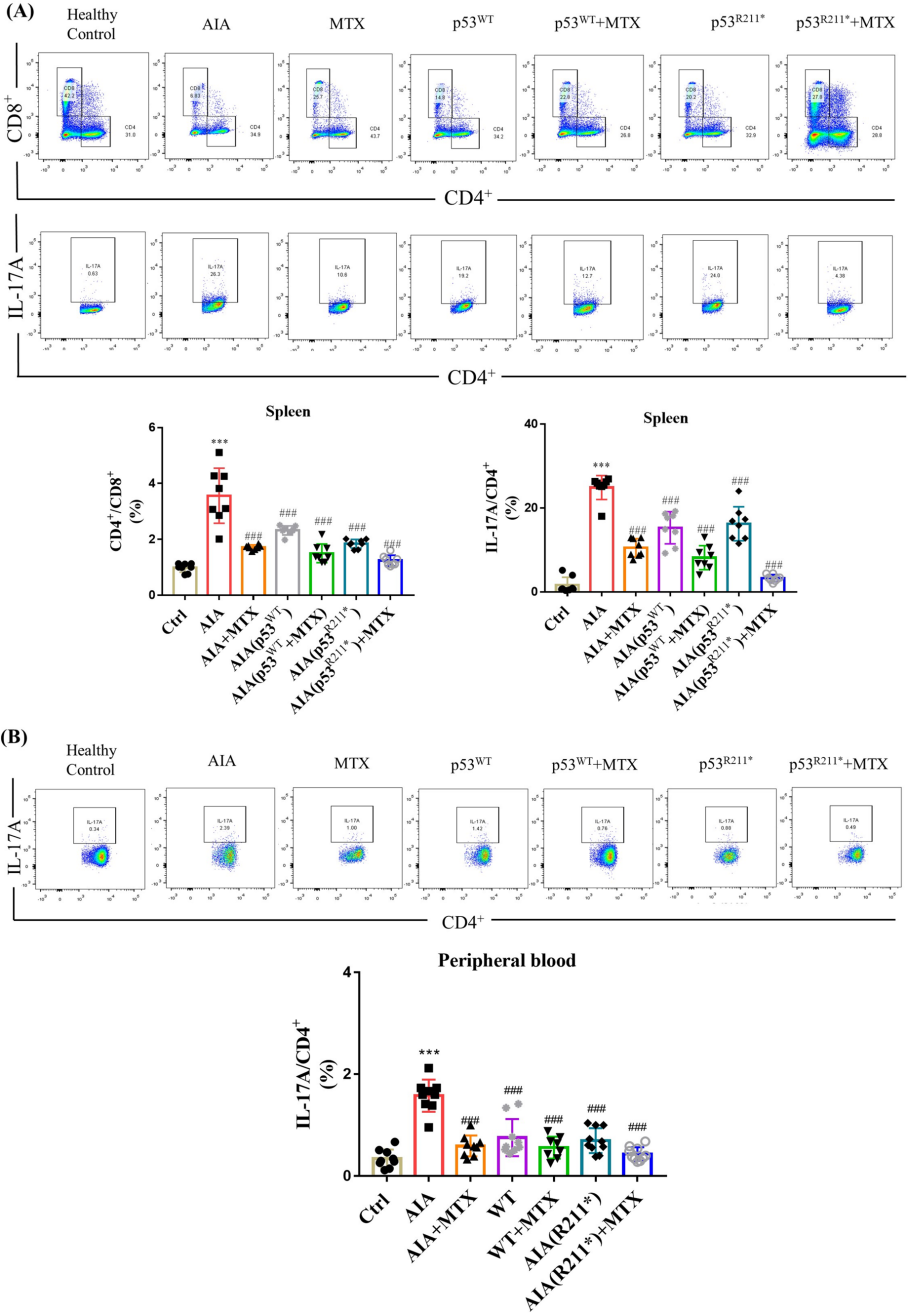
The immunomodulatory effect of AAV-p53^R211*^ in AIA rats by single intra-articular knee injection. **A**, **B** Immunological impact of articular knee injection of AAV-p53^R211*^ in AIA model. The AIA rats from 6 model groups were articular knee injected with AAV-EGFP, AAV-p53^WT^ or AAV-p53^R211*^ (1 × 10^11^ PFU) with or without MTX treatment and monitored for 27 days. Peripheral blood and spleen lymphocytes were then harvested from these rats for flow cytometry analysis of T cell activation using fluorescent antibodies against CD45, CD3, CD4, CD8, Foxp3 and IL-17A. Representative scatter plot images displayed the CD4^+^ T cells under Th17 cell differentiation conditions. The quantitative bar charts show the percentage of CD8^+^ cells among CD4^+^ T lymphocytes and the percentage of IL-17A cells among CD4^+^ T lymphocytes. The data are expressed as mean ± SEM (*n* = 6–8) from three independent experiments. One-way ANOVA; ###*p* < 0.001 compared with AIA control group; ***p < 0.001 compared with healthy controls

To examine the autoimmune response in AAV-mediated overexpression of p53^R211*^ mutant in AIA rats, CD4^+^ and CD8^+^ T lymphocytes were isolated from spleen and total peripheral blood lymphocytes using antibodies against CD3^+^. Compared with AAV-EGFP-injected AIA rats, the ratio of CD4^+^/CD8^+^ and the percentage of Th17 T cells characterized by IL-17A production were declined remarkably in the splenocytes of AAV-p53^WT^ or AAV-p53^R211*^-injected AIA rats (*p* = 0.0002), and the results were comparable to the MTX-treated group (Fig. 3A). However, an insignificant fluctuation in the population of Treg cells, where Foxp3 served as a lineage specification factor, was observed in AAV-p53^WT^ or AAV-p53^R211*^-injected group as well as other AIA groups (Supplementary Figure S1). Consistently, the population of Th17 lymphocytes in total peripheral blood of AAV-p53^WT^ or AAV-p53^R211*^-injected AIA rats was significantly lower than that in AAV-EGFP-injected AIA rats (*p* < 0.0001) (Fig. 3B), whereas there was no obvious change in the population of Treg cells in all treatment groups (Supplementary Figure S1). These results demonstrated that the activation of CD4^+^ and differentiation of Th17 lymphocytes were retarded by overexpression of p53^R211*^ mutant in AIA rat model. Of note, AAV-p53^WT^-injected group also exhibited similar immunomodulatory effects on CD4^+^ and Th17 cells but to a lesser extent. Accordingly, the overexpression of p53^R211*^ mutant attenuated the autoimmune response in AIA rats mainly via suppressing the activation of CD4^+^ lymphocyte and the differentiation of Th17 cells.

### p53^R211*^ mutant suppressed the release of pro-inflammatory cytokines and reduced synovial hyperplasia in AIA rats

During the pathogenesis of RA, Th17 cells produce various pro-inflammatory cytokines such as IL-17A and Granulocyte–macrophage Colony Stimulating Factor (GM-CSF), whereas T helper 1/2 cell (Th1/Th2) cells secret Interferon-γ (IFN-γ) and Interleukin-6 (IL-6), thereby promote synovitis [46]. In addition, the infiltration of pathogenic immune cells into joint together with the excessive release of proinflammatory cytokines promote the proliferation of synovial fibroblasts and the destruction of cartilage and bone [47, 48].

**Fig. 4:**
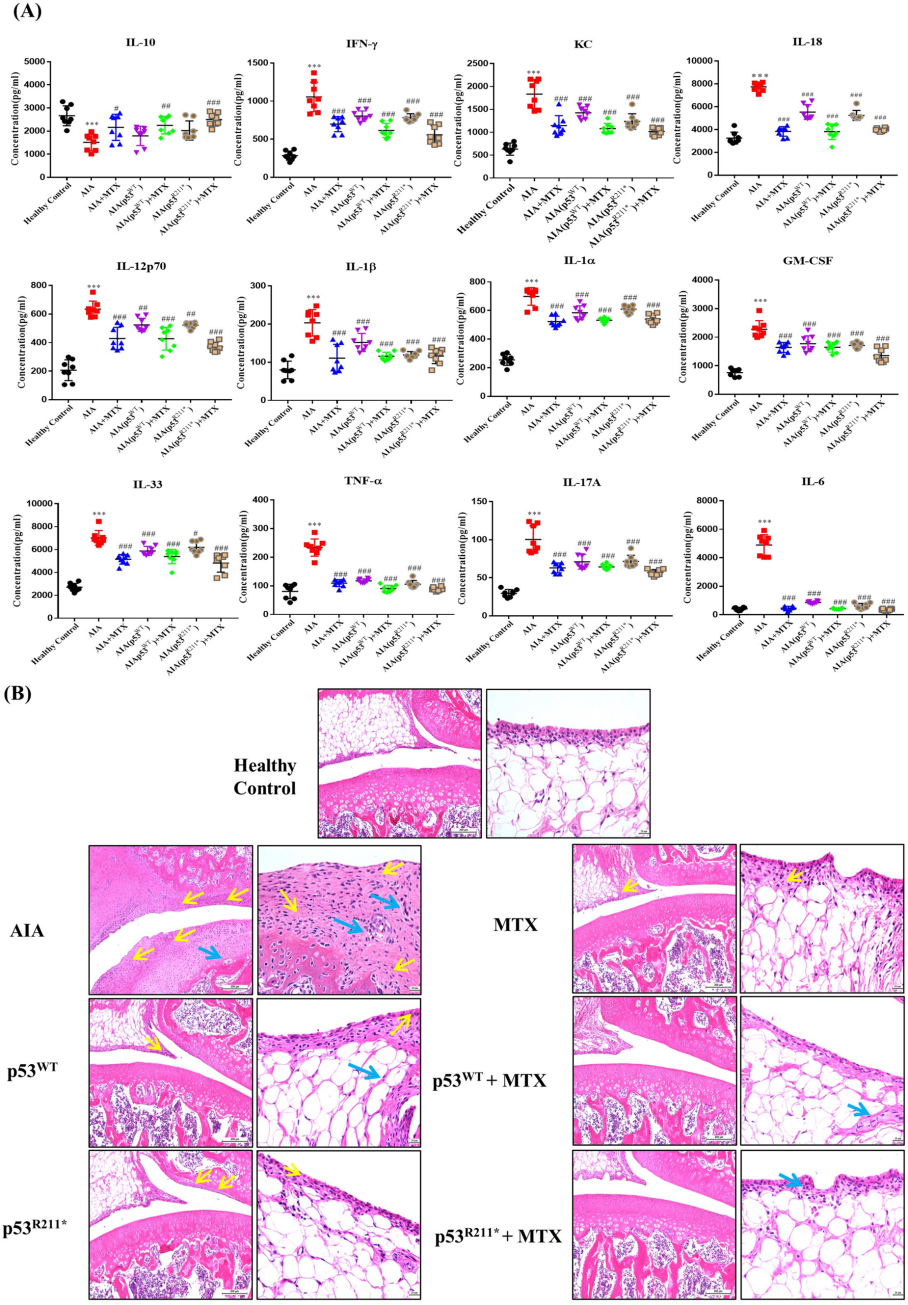
The anti-inflammatory effects of AAV-p53^R211*^ in AIA rats by single articular knee injection. **A** The T-helper-specific multiplex cytokine profile of rat blood sample. Serums were harvested from rat blood and analyzed by flow cytometry for the expression of inflammatory cytokines. **B** Representative histological sections of synovial membranes stained with H&E (scale bar: left 200 µm and right 20 µm). Representative images are shown with the same magnification. Yellow arrows indicate the synovial lining layer containing the hyperplasia of neutrophils, lymphocytes, plasma cells and other inflammatory cells; blue arrows indicate the proliferation of interstitial fibroblasts with neovascularization. Data are shown as mean ± SEM (*n* = 6–8). One-way AVOVA; ^#^*p* < 0.05, ^##^*p* < 0.01, ^###^*p* < 0.001 compared with AIA control group, ***p < 0.001 compared with healthy controls (color figure online)

In current study, we quantified the 12 representative cytokines by using of a multiplex panel to examine the proinflammatory effect of p53^R211*^ mutant in AIA rats (Fig. 4A). Results demonstrated that the pro-inflammatory cytokines such as Tumor Necrosis Factor alpha (TNF-α), IL-6, IFN-γ, Interleukin 1β (IL-1β), GM-CSF and IL-17A were significantly declined (*p* < 0.0001), while the expression of anti-inflammatory cytokine Interleukin 10 (IL-10) was significantly elevated in AAV-p53^R211*^-injected AIA rats compared to AIA control rat (*p* = 0.0193). Similar trends were also observed in AAV-p53^WT^-injected AIA rats but generally to a lesser extent. Notably, co-treatment with MTX in AAV-p53^WT/R211*^-injected AIA rats further reduced the expression of pro-inflammatory cytokines and elevated IL-10 expression to the level similar to those of MTX-treated rats, implying that no detrimental effect of p53 wide type or R211* mutant was observed in the MTX treatment.

Hematoxylin–eosin (H&E) staining of joint sections was also performed to evaluate the inflammatory conditions of synovial membrane in AAV-p53^R211*^-injected rats (Fig. 4B). Synovial hyperplasia (yellow arrow) with infiltrations of neutrophils, T lymphocytes, plasma cells and macrophages were found, whereas proliferation of interstitial fibroblasts (blue arrow) with neovascularization and destruction of cartilage were commonly observed in AIA rats compared with healthy controls. Apparently, both AAV-p53^R211*^ and -p53^WT^ injections alleviated the histological damaging phenotypes in AIA rats (Fig. 4B). In similar fashion, single or co-treatment with MTX also relieved such damage as observed in Fig. 4B. These results revealed that the overexpression of p53^R211*^ mutant repressed the production of pro-inflammatory cytokines along with the increase of anti-inflammatory cytokine expression, thus ameliorating synovial hyperplasia in AIA rats.

### p53^R211*^ mutant attenuated the arthritic condition predominantly via regulation of immunity pathways in AIA rats

To further elucidate the underlying mechanisms of p53^R211*^ mutant-mediated anti-arthritic effect in AIA rats, we analyzed and compared the differentially expressed genes (DEGs) in knee joint of AAV-p53^R211*^- and AAV-EGFP-injected AIA rats using total RNA sequencing (RNA-Seq).

**Fig. 5:**
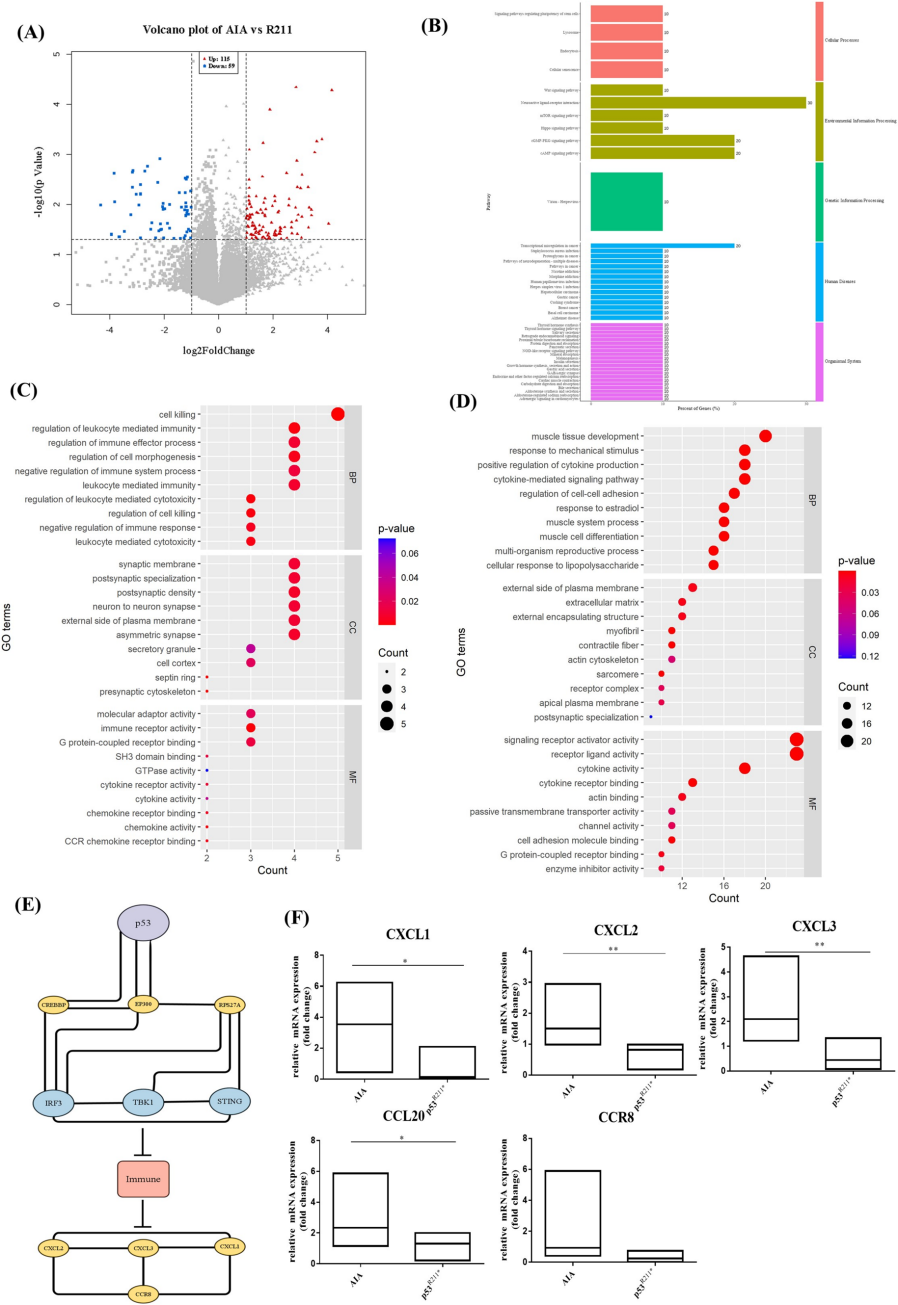
Differentially expressed genes (DEGs) for AAV-mediated overexpression of p53^R211*^ in AIA rats are enriched in the autoimmune pathways as analyzed by RNA sequencing. **A** Volcanic map of differentially expressed mRNAs. Red dots represent significantly upregulated genes and blue dots represent significantly downregulated genes. **B** KEGG classification chart constructed from KEGG orthology (KO) databases using DEGs. Length of chart represent gene rations of DEGs participated in each of the pathway and labeled at the right of each chart. The color of the charts represents classification provided by KO analysis results. **C**, **D** Bubble diagram for the Gene Ontology (GO) analysis of DEGs. Biological processes are ranked on the enrichment fraction of each genome. The color of the bubbles represents *p* value. The size of the bubbles represents the gene enrichment ratio. **E** Protein–Protein interaction networks functional enrichment analysis analyzed by STRING and reorganized by Cytoscape (version 3.9.1). **F** Gene verification of the mRNA sequencing data by RT-qPCR. The 5 genes related to inflammatory chemokines, including CXCL1, CXCL2, CXCL3, CCL20 and CCR8, were selected for qPCR analysis. Gene expression was normalized to b-actin relative to the AIA vehicle control and analyzed using 2^−ΔΔCT^. Data are presented as mean ± SEM. One-way AVOVA; *p < 0.05, **p < 0.01, significantly different from AIA control group (color figure online)

Volcano plot analysis was first performed to identify DEGs from the differences between AAV-p53^R211*^- and AAV-EGFP-injected AIA rats. As shown in Fig. 5A, 174 DEGs comprising 115 upregulated (red) and 59 downregulated (blue) genes were identified with *p* value less than 0.05 and fold change greater than 2. KEGG analysis indicated that 47 pathways determined from these genes were associated with immune response (Fig. 5B). In addition, GO enrichment analysis revealed that the molecular functions and cellular components such as cell killing, regulation of leukocyte mediated immunity, regulation of cell morphogenesis, regulation of immune effector process and leukocyte mediated immunity were significantly enriched by the upregulated DEGs (Fig. 5C), whereas cytokine-mediated signaling pathways and cellular responses to lipopolysaccharide were markedly enriched by the downregulated DEGs (Fig. 5D). Of note, STRING analysis recognized 11 genes from the protein–protein interaction network, in which 3 genes TANK-binding kinase 1 (TBK1), Interferon regulatory factor 3 (IRF3) and Stimulator of interferon genes (STING) interact together as the key role of immune response activation (Fig. 5E). Importantly, previous studies reported that mutant p53 could inhibit innate immune TBK1-IRF3-STING cascade via direct binding to TBK1 [18], proposing that p53^R211*^ mutant may interact with TBK1-IRF3-STING signaling cascade via a similar mechanism. Notably, this innate immune pathway is responsible for the activation and the release of various chemokines and cytokines [49, 50]. Subsequently, five representative chemokines, C-X-C Motif Chemokine 1/2/3 (CXCL1, CXCL2, CXCL3), chemokine (C–C motif) ligand 20 (CCL20) and CC chemokine receptor (CCR8), identified from DEGs with greater differential expression were validated by qPCR. As shown in Fig. 5F, the mRNA expression levels of all these chemokines were significantly suppressed in AAV-p53^R211*^-injected group in comparison with AAV-EGFP-injected AIA control rats (*p* = 0.028). These results indicated that the anti-arthritic effect of p53^R211*^ may act through the inactivation of immune-related pathways especially TBK1-IRF3-STING signaling cascade, thereby inhibiting the expressions of downstream chemokines (Fig. 5F) and pro-inflammatory cytokines (Fig. 4A).

### p53^R211*^ suppresses innate immune response via direct inhibition of TBK1-IRF3-STING signaling pathway

Based on the analysis of RNA-Seq results in Fig. 5A–E, we hypothesized that p53^R211*^ mutant possibly interacts with immune-related genes TBK1, IRF3 and STING to ameliorate the immune and inflammatory responses in AIA rats. Recent studies reported that mutant p53 hindered TBK1-IRF3-STING trimeric complex formation by direct binding to TBK1, and subsequently blocked IRF3 nuclear translocation to intervene immune signaling [18]. Hence, we postulated that the immunomodulatory effect of p53^R211*^ may proceed through a similar mechanistic pathway.

**Fig. 6:**
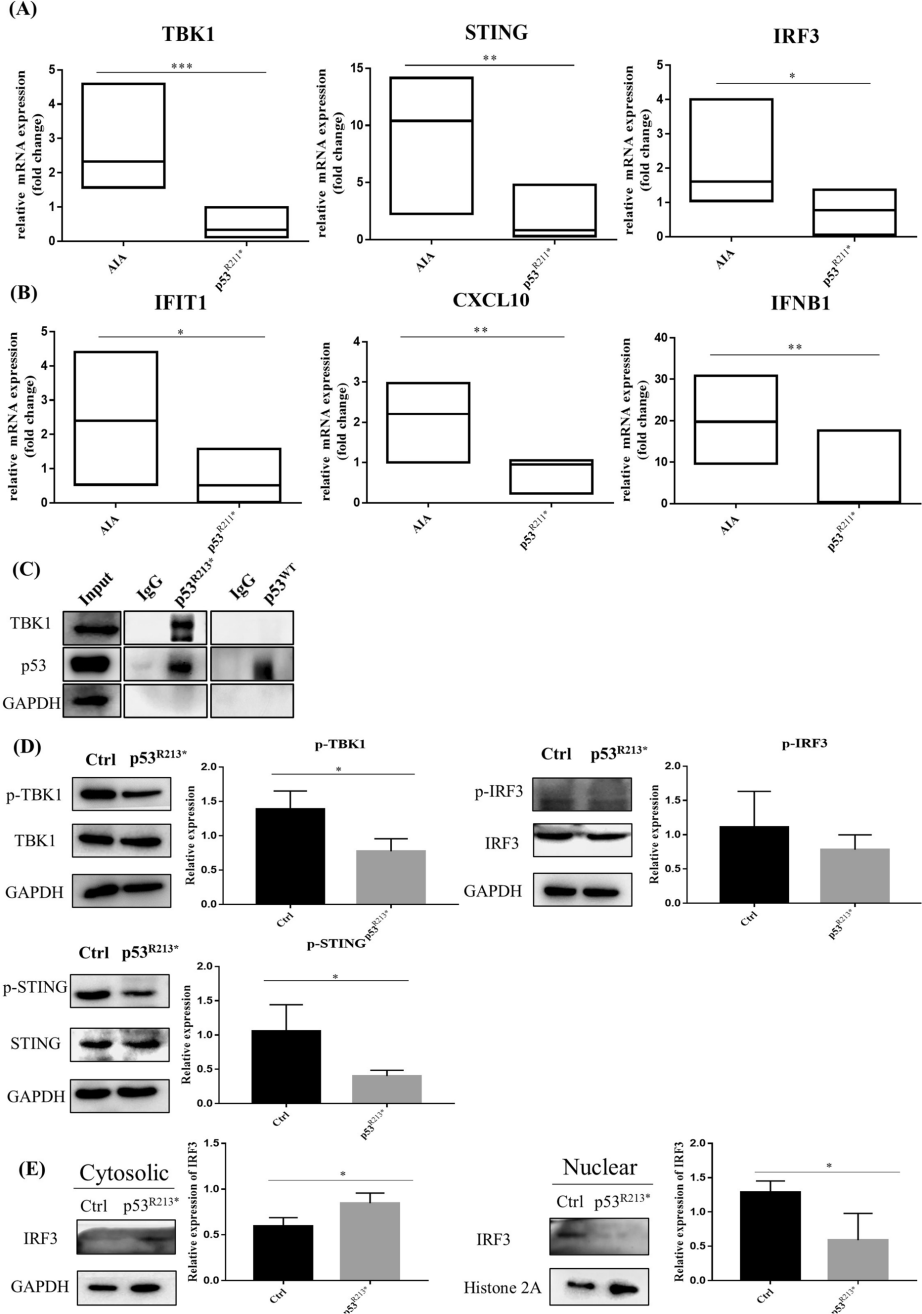
Overexpression of p53^R211*^ suppresses innate immunity via intervention of TBK1-IRF3-STING signaling cascade. **A** RT-qPCR analysis of innate immune-related genes (TBK1, STING and IRF3) in the synovium of AIA rats with or without AAV-p53^R211*^ injection. **B** The mRNA expressions of various IRF3-controlled inflammatory cytokines (IFIT1, CXCL10 and IFNB1) in the synovium of AAV-p53^R211*^- and AAV-EGFP-injected AIA rats. **C** The interaction of human p53^R213*^ mutant with TBK1 protein in RAFLS. The p53^R213*^ and p53^WT^ plasmid DNA were transfected into RAFLS cell line for 48 h and was immunoprecipitated from whole cell lysates. The cell lysates and immunoprecipitated proteins (IP) were then analyzed by Western blotting. **D** Overexpression of human p53^R213*^ mutant suppressed the phosphorylation of TBK1, IRF3 and STING in RAFLS. RAFLS were transfected with p53^R213*^ plasmid DNA for 48 h. Cell lysates were collected and analyzed by Western blotting using antibodies against p-TBK1, p-IRF3 and p-STING. The bar charts show the quantification of target protein expressions versus GAPDH expression from three independent experiments using ImageJ software. **E** Western blot analysis of IRF3 expression in cytoplasmic and nuclear fractions from RAFLS with or without the overexpression of p53^R213*^ mutant. GAPDH and Histone 2A were used as loading controls for the cytoplasmic and nuclear fractions, respectively. *p < 0.05, **p < 0.01, ***p < 0.001 compared with AIA control group or mock transfected control cells

To verify the correlation of p53^R211*^ mutant with TBK1-IRF3-STING signaling pathway, the expression of these three genes in rat synovial membrane was assessed by qPCR. As shown in Fig. 6A, AIA rat injected with AAV-p53^R211*^ demonstrated a significant decrease in the mRNA expressions of TBK1, IRF3 and STING in comparison with the AIA control (*p* < 0.0001). We then examined the expressions of IRF3 downstream target genes such as Interferon induced transmembrane protein 1 (IFIT1), -X-C Motif Chemokine 10 (CXCL10) and Interferon beta 1 (IFNB1), and the results showed that the expression levels of these genes were remarkably suppressed in AAV-p53^R211*^-injected AIA rats (*p* = 0.011) (Fig. 6B).

With referenced to the above in vivo data, we further explored the interaction of p53 mutant with TBK1 in RAFLS using the corresponding human p53^R213*^ mutant construct. Consistent with the results reported in literature [18], immunoprecipitation illustrated that only p53^R213*^ mutant was bound with TBK1 instead of the p53^WT^ (Fig. 6C). Of note, overexpression of p53^R213*^ mutant also inhibited the phosphorylation of TBK1, IRF3 and STING in RAFLS (Fig. 6D), and these findings were coincided with the in vivo results in Fig. 6A. Furthermore, overexpression of p53^R213*^ mutant markedly reduced the nuclear localization of IRF3 in RAFLS, thus retaining the expression of IRF3 in the cytoplasm (*p* = 0.0406) (Fig. 6E). These findings proposed that rat p53^R211*^ mutant (human p53^R213*^ mutant) directly binds and interacts with TBK1 to hamper the formation of trimeric TBK1-IRF3-STING complex and the subsequent nuclear localization of IRF3, thereby suppressing the immune response. It also well explained that the immunosuppressive effect of p53^R211*^ mutant is superior to p53 wild type as observed in our in vivo study (Fig. 3A, B).

### Overexpression of p53^R211*^ suppresses the phosphorylation of TBK1, IRF3, STING and inhibits the infiltration of Th17 cells

**Fig. 7:**
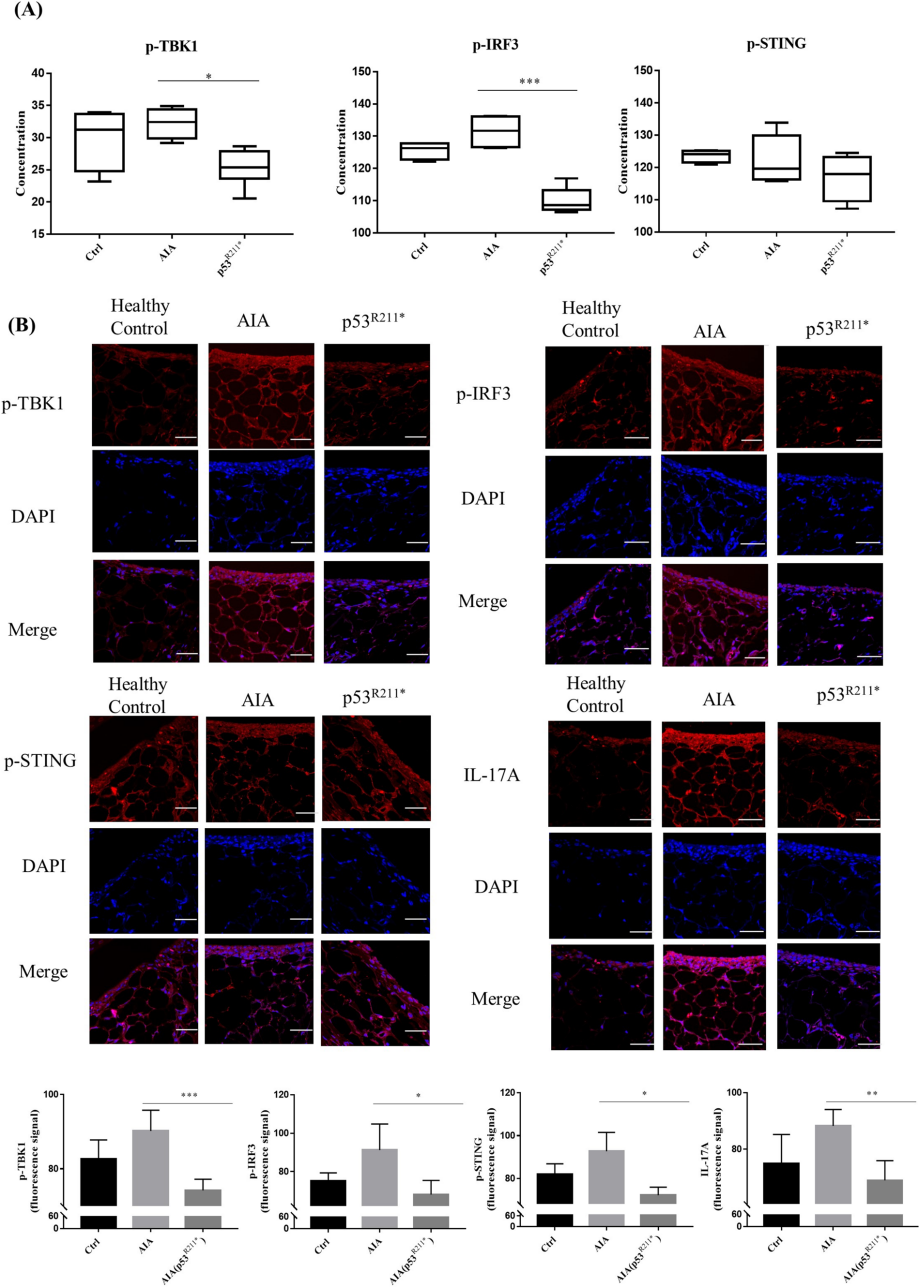
Overexpression of rat p53^R211*^ mutant hinders TBK1-IRF3-STING trimeric complex formation and specifically reduces IL-17A expression in vivo. **A** Detection of p-TBK1, p-IRF3 and p-STING concentration in blood serum of the above treatment groups by ELISA assay. Blood serum was harvested from healthy controls, AAV-p53^R211*^- and AAV-EGFP-injected AIA rats. *p < 0.05, **p < 0.01, ***p < 0.0001 compared with AIA control group. **B** Representative immunofluorescence images (scale bar: 38.6 µm) demonstrate the synovial tissues from healthy controls, AAV-EGFP-injected and AAV-p53^R211*^-injected AIA rats (*n* = 5). Synovial tissues from five rats were sectioned and immune-stained with antibodies against p-TBK1, p-IRF3, p-STING and IL-17A prior to the treatment with anti-rabbit 2nd antibody conjugated with TRITC. The bar charts show the quantitative analysis of the immunofluorescent signals of the proteins by ImageJ software

Following the promising results obtained from the mutant p53^R213*^-mediated blockage of TBK1-IRF3-STING immune pathway in RAFLS, we further determined the concentrations of p-TBK1, p-IRF3 and p-STING in the blood serum of AAV-p53^R211*^-injected AIA rats by ELISA assay. Consistent with the gene expression analysis in synovial tissue, the concentrations of these phosphorylated proteins were markedly reduced in the serum of AAV-p53^R211*^-injected AIA rats when compared with AIA control (*p* = 0.0009) (Fig. 7A). Concomitantly, immunofluorescence (IF) staining also confirmed that the expression levels of p-TBK1, p-IRF3 and p-STING in the synovial tissue of AAV-p53^R211*^-injected AIA rats were significantly down-regulated in comparison with AIA control (*p* = 0.0001) (Fig. 7B). Our findings revealed that the overexpression of p53^R211*^ mutant exhibited an anti-inflammatory effect mainly through the suppression of innate immune TBK1-IRF3-STING cascades.

T lymphocytes take a major role in both RA pathogenesis and tumor immunity. A recent study demonstrated that p53^R249S^ mutant reduced the infiltration of CD4^+^ T cells in tumor tissue, mainly via the suppression of innate immune TBK1-IRF3-STING pathway [18]. This eventually suppressed the IRF3-mediated downstream Th17 responses and the subsequent production of inflammatory cytokine IL-17A [51–53]. Coincidently, our results indicated that the population of Th17 cells was significantly suppressed in both splenocytes and peripheral blood cells from AAV-p53^R211*^-injected AIA rats (Fig. 3A and B). Accordingly, we further examined the infiltration of Th17 cells in synovial tissue by detection of IL-17A (Fig. 7B). Consistently, AAV-mediated overexpression of p53^R211*^ reduced the IL-17A expression in synovial tissue of AIA rat, suggesting the blockage of infiltration of Th17 cells. In conclusion, our results indicated that p53^R211*^ mutant ameliorated the arthritic condition in AIA rat and prevented the infiltration of Th17 cells in synovial tissue possibly through the direct intervention of TBK1-IRF3-STING immunological cascades.

## Discussion

In this study, we observed the experimental differences, in which p53 mutant exhibits immunomodulatory effect without resistance to MTX in vivo (Figs. 2 and 3) but not observed in vitro (Supplementary Table S1). Rheumatoid arthritis fibroblast-like synoviocyte (RAFLS) derived from synovial tissue of RA patients has been widely used as a representative cell line for studying inflammation, apoptosis and cell proliferation in RA research [54]. Although RA is symptomized by chronic inflammation in joint, its pathogenesis originates from systemic immune abnormality [55]. Therefore, using a single in vitro cell model alone is inadequate to simulate the complicated in vivo immune environment of RA [56]. Discrepancies are commonly observed between in vitro and in vivo studies, especially in immunological studies [57]. For instance, bone marrow-derived mesenchymal stem cells (MSC) suppressed T cell proliferation in vitro, while MSC treatment did not show immunomodulatory effect via inhibiting T cell proliferation nor ameliorate the disease severity in collagen-induced arthritis (CIA) mice [58]. Another study demonstrated that CD3-peptides had no effect on T cell function in vitro but unexpectedly repressed inflammatory responses in AIA rats [56]. Moreover, adiponectin (AD) had an obvious anti-inflammatory function in CIA mice [59], but previous data suggested that AD increased IL-6 expression in synovial fibroblasts [60]. Such experimental discrepancies were also observed in other diseases related immune dysfunction such as diabetes mellitus. Diabetic db+/db+ mice showed defects in immune functions in terms of delayed skin graft rejection and a significant increase in plaque-forming cell (PFC) response to sheep erythrocytes (SRBC) [61]. However, contrary in vitro results were obtained with spleen cells isolated from those db+/db+ mice, where negligible fluctuations were observed in responses to allogeneic cells and SRBC-induced PFC. Other than the intricate immune responses, stiffness of cell environment affects biomechanical and physiochemical properties of cells, becoming another factor for in vitro and in vivo discrepancies [62]. Together with these cited examples, our study well explain the importance of in vivo study that provides a more comprehensive view for examining disease pathogenesis, although in vitro studies are still irreplaceable due to low cost and high efficiency.

Apart from p53^R213*^ mutant, p53^WT^ also exhibited a similar anti-inflammatory effect in AIA rats (Fig. 2A–C); however, p53^WT^ showed no interaction with TBK1 (Fig. 6C), implying a different anti-inflammatory mechanism. In fact, p53^WT^ has been extensively reported to regulate the expressions of inflammatory cytokines and Matrix metalloproteinases (MMPs) via various pathways [63]. Overexpressed p53^WT^ suppressed the expression of IL-6 in AIA rats, via inactivation of nuclear factor kappa-B (NF-κB), Mitogen-activated protein kinase (MAPK) and Extracellular regulated protein kinases (ERK) pathways [64]. Specific dephosphorylation of p38 MARK by WIP-1 phosphatase was mediated by p53^WT^ [65]. Moreover, overexpression of p21, the downstream gene of p53^WT^, also declined the expressions of IL-6 and MMP-1 in RAFLS, via Jun kinase/Activator protein-1 (JNK/AP-1) pathway [66]. Apparently, in vivo study further proved that p53-deficient mice model exacerbated the severity of arthritis with increasing expressions of collagenase-3 and pro-inflammation cytokines [67]. Meanwhile, p53 function is highly associated with the homeostasis of innate and adaptive immune responses. Deficiency of p53 in RA downregulated Toll-like receptors expressions and modulates T cell differentiation, and ultimately triggered chronic inflammation [63]. This proposes that p53^R213*^ mutant exhibits anti-inflammation in RA, independent of p53^WT^-mediated pathway.

As a central mediator of innate immune defense system, TBK1 activates downstream genes for production of type I interferons (IFNs) and other inflammatory cytokines against pathogenic infection [68]. However, overactivation of TBK1 is closely related to dysregulation of immune responses, which is key pathogenesis of autoimmune diseases [69]. For example, elevated TBK1 expression was commonly observed in autoimmune diseases including systemic lupus erythematosus (SLE) [70], primary Sjögren’s syndrome (pSS) and systemic sclerosis (SSc) patients [71]. Patients with RA also showed upregulation of TBK1 downstream IFN-I expression [72]. Similarly, our results also revealed a high expression level of pTBK1 (Fig. 7A), overactivation of CD4^+^ cells (Fig. 3A) and infiltration of Th17 cells (Fig. 7B) in AIA rats. Likewise, TBK1-deficient FLS revealed the suppression of IFN-β and IP-10 expressions [73]. All these results suggest the pathogenic role of TBK1 in autoimmune diseases including RA, thus proposing TBK1 as a potential therapeutic target. Indeed, the use of pyrimidine-based TBK1 inhibitor was found to downregulate IFN expression in vitro and in vivo [74]. Another study also reported that TBK1 inhibitor greatly delayed the onset and decreased the severity of experimental autoimmune encephalomyelitis (EAE), and even suppressed the relapse of EAE [75]. Moreover, treatment with TBK1/IKKε inhibitor reduced IFN-I level in PBMCs extracted from pSS, SLE and SSc patients [71]. Herein, we also reported that binding of TBK1 with p53^R213*^ mutant inhibited TBK1-STING-IRF3 cascade, thereby exhibiting immunosuppressive and anti-inflammatory effects in AIA rats. These results well demonstrate TBK1 as a promising drug target for RA, although no TBK1 inhibitor has been approved for RA treatment [76]. Therefore, the development of small molecules or small peptides as TBK1 inhibitor provides a prospective strategy for the treatment of RA or even other autoimmune diseases.

P53 mutants, such as R248W, R249S, R273H and R280K, have been reported to intervene cyclic GMP-AMP synthase (cGAS)-STING-TBK1-IRF3 immune cascade, resulting in immune evasion and thus promoting tumorigenesis in cancer[18]. Similarly, we also clarified that human p53^R213*^ (rat p53^R211*^) suppressed immune response in RA mainly via blocking TBK1-IRF3-STING innate immune pathway. Although variable outcomes of p53-mediated immune response in cancer and RA development were observed, we propose that the effect of p53 mutants on immunity may share similar mechanism. For instance, mutant p53 correlated with upregulation of programmed cell death-Ligand 1 (PD-L1) which facilitated cancer cells to escape from immune system [77, 78]. However, in autoimmune disease, PD-L1 showed an opposite outcome by suppressing autoreactive T cell function [79]. Another representative example is that mutant p53 compromises the transcription of immune-related Toll-like-receptor 3 (TLR3) in cancer cells [80, 81]. And conversely, inhibition of TLR3 attenuates the symptom of RA [82]. Of note, TLR3 activation is responsible for inflammatory status, promotion of osteoclast differentiation and expressions of B cell survival/proliferating factors B cell activating factor (BAFF) and aspartate aminotransferase-to-Platelet Ratio Index (APRI) in RA [83–85]. Moreover, mutant p53 induces suppression of macrophage activation and promotes immunosuppressive Treg cell via expression of Transforming growth factor beta (TGF-β), thereby facilitating tumor progression [86]. Conversely, in RA condition, macrophages are usually over-activated, and the differentiation and function of Treg cells are commonly suppressed [87]. One more example is that antigen presentation triggers abnormal differentiation of autoreactive T cells and accelerates autoimmune response in RA [88, 89]. Alternatively, major histocompatibility complex class I (MHC-I) related antigen presentation was retarded by mutant p53 in cancer [78]. In brief, mutations of p53 promote cancer pathogenesis by mediating immune evasion and proliferation of tumor cells, while these immune escape mechanisms may impose opposite consequences in RA. We here demonstrated that human p53^R213*^ (rat p53^R211*^) attenuated the disease severity mainly by its immunomodulatory effect in RA. In addition to the blockage of TBK1-IRF3-STING pathway, mutant p53^R211*^ may exhibit immunomodulatory effect through aforementioned mechanism. Given that the effect of different p53 mutations on RA pathogenesis has not been well explored so far, further studies are required to unravel the detailed mechanism of action.

### Supplementary Information

Below is the link to the electronic supplementary material.

Supplementary file1 (DOCX 3182 KB)

## Data Availability

RNA sequencing data generated in this study has been deposited in GEO (GSE221158). The following secure token has followed this link as https://www.ncbi.nlm.nih.gov/geo/query/acc.cgi?acc=GSE221158. The secure token for accessing GSE221158: kvwxsmwshpkrpgz.
