# Supplementary material for: Mutant p53R211* ameliorates inflammatory arthritis in AIA rats via inhibition of TBK1-IRF3 innate immune response
Source: Inflamm Res. 2023 Nov 8;72(12):2199–219. doi: 10.1007/s00011-023-01809-w (PMC10656327; doi:10.1007/s00011-023-01809-w)
Supplement: Supplementary file 1 — Supplementary file1 (DOCX 3182 KB) [file 11_2023_1809_MOESM1_ESM.docx]

Supplementary Material

**Mutant p53^R211*^ ameliorates inflammatory arthritis in AIA rats *via* inhibition of TBK1-IRF3 innate immune response**

**Yaling Zeng^1^, Jerome P. L. Ng^1^, Linna Wang^1^, Xiongfei Xu^1^, Betty Yuen Kwan Law^1^, Guobing Chen^2^, Hang Hong Lo^1^,** **Lijun Yang^1^, Jiujie Yang^1^, Lei Zhang^1^, Liqun Qu****^1^, Xiaoyun Yun^1^, Jing Zhong^1^, Ruihong Chen^1^, Dingqi Zhang^1^,** **Yuping Wang^1^, Weidan Luo^1^,** **Congling Qiu^2^, Baixiong Huang^1^, Wenfeng liu^3^, Liang Liu^1*^,** **Vincent Kam Wai Wong^1*^**

^1^Dr. Neher’s Biophysics Laboratory for Innovative Drug Discovery, State Key Laboratory of Quality Research in Chinese Medicine, Macau University of Science and Technology, Macau 999078, China

^2^Department of Microbiology and Immunology, Institute of Geriatric Immunology, School of Medicine, Jinan University, Guangzhou 510630, China

^3^School of Biotechnology and Health Sciences, Wuyi University, Jiangmen 529020, China

*** Corresponding authors:**Vincent Kam Wai Wong
bowaiwong@gmail.com

Liang Liu

lliu@must.edu.mo

Tel: +853-8897-2408 (VKWW); +853-8897-2238 (LL)

Fax: +853-2882-2799 (VKWW); +853-2882-3312 (LL)

Context

[1 Supplementary Data 2](#_Toc127799169)

[1.1 Supplementary Figures 2](#_Toc127799170)

[1.2 Supplementary Tables 2](#_Toc127799171)

[1.3 Original western blot for three repeats 5](#_Toc127799172)

# Supplementary Data

## Supplementary Figures


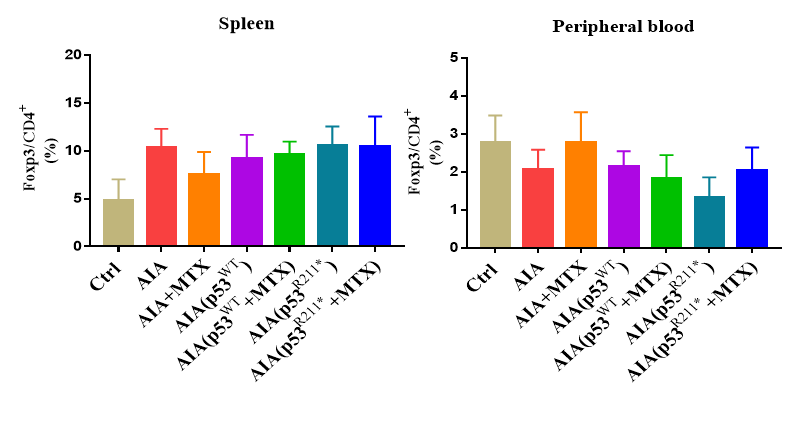

**Supplementary Figure S1.** The immunomodulatory effect of intra-articular injection of AAV-p53^R211*^ on Treg cells in AIA rats. Flow cytometric analysis of Treg cells from rat peripheral blood and spleen of seven animal groups. Treg cells were identified by intracellular staining of anti-Foxp3 antibody. Foxp3^+^ Tregs were gated on CD4^+^ T cell. The bar charts show the percentage of Foxp3 among CD4^+^ T lymphocytes. The data are expressed as mean ± SEM (n =6-8). The statistical analysis is performed by one-way ANOVA.

## Supplementary Tables

**Supplementary Table S1:** **p53 mutants cause HCQ/FK506/CSA/LEF-resistant phenotype in RAFLS.**

| Mutant | HCQ IC_50_ | | | RF | FK506 IC_50_ | | | RF | CSA IC_50_ | | | RF | LEF IC_50_ | | | RF |
| --- | --- | --- | --- | --- | --- | --- | --- | --- | --- | --- | --- | --- | --- | --- | --- | --- |
| CTRL | 46.83 | ± | 5.52 |  | 71.10 | ± | 3.05 |  | 16.53 | ± | 1.75 |  | 527.67 | ± | 36.25 |  |
| WT | 40.00 | ± | 3.86 | 0.85 | 67.13 | ± | 10.22 | 0.94 | 15.87 | ± | 3.03 | 0.96 | 516.67 | ± | 41.67 | 0.98 |
| V73M | 58.97 | ± | 17.21 | 1.47 | 58.20 | ± | 3.47 | 0.87 | 20.33 | ± | 0.58 | 1.28 | 456.33 | ± | 30.92 | 0.88 |
| P75L | 35.80 | ± | 0.35 | 0.90 | 97.00 | ± | 1.23 | 1.44 | 18.93 | ± | 0.58 | 1.19 | 930.67 | ± | 50.29 | 1.80 |
| S99P | 31.13 | ± | 0.38 | 0.78 | 82.97 | ± | 0.95 | 1.24 | 22.03 | ± | 1.33 | 1.39 | 669.00 | ± | 31.43 | 1.29 |
| K101E | 37.47 | ± | 0.68 | 0.94 | 77.20 | ± | 9.66 | 1.15 | 12.50 | ± | 1.00 | 0.79 | 636.33 | ± | 4.16 | 1.23 |
| A119T | 80.47 | ± | 0.80 | 2.01 | 74.70 | ± | 4.19 | 1.11 | 24.20 | ± | 3.86 | 1.53 | 770.33 | ± | 8.14 | 1.49 |
| K120E | 29.87 | ± | 1.80 | 0.75 | 79.70 | ± | 3.50 | 1.19 | 14.00 | ± | 1.78 | 0.88 | 934.00 | ± | 52.00 | 1.81 |
| S121P | 76.83 | ± | 4.72 | 1.92 | 148.67 | ± | 9.29 | 2.21 | 13.87 | ± | 0.21 | 0.87 | 341.67 | ± | 6.11 | 0.66 |
| T123A | 32.83 | ± | 1.04 | 0.82 | 78.17 | ± | 1.88 | 1.16 | 10.87 | ± | 0.12 | 0.68 | 642.00 | ± | 25.36 | 1.24 |
| T125M | 32.27 | ± | 0.76 | 0.81 | 98.27 | ± | 1.33 | 1.46 | 13.07 | ± | 1.75 | 0.82 | 671.33 | ± | 25.15 | 1.30 |
| P128T | 28.07 | ± | 1.50 | 0.70 | 12.43 | ± | 0.91 | 0.19 | 31.13 | ± | 1.03 | 1.96 | 978.33 | ± | 26.31 | 1.89 |
| K132E | 32.10 | ± | 1.08 | 0.80 | 116.67 | ± | 7.77 | 1.74 | 12.07 | ± | 0.95 | 0.76 | 570.33 | ± | 62.01 | 1.10 |
| F134L | 33.97 | ± | 0.06 | 0.85 | 106.33 | ± | 0.58 | 1.58 | 26.47 | ± | 4.15 | 1.67 | 635.67 | ± | 24.83 | 1.23 |
| A138V | 26.07 | ± | 3.88 | 0.65 | 113.67 | ± | 0.58 | 1.69 | 34.00 | ± | 2.40 | 2.14 | 704.67 | ± | 5.69 | 1.36 |
| A138T | 25.60 | ± | 6.39 | 0.64 | 54.27 | ± | 1.10 | 0.81 | 19.00 | ± | 1.57 | 1.20 | 743.67 | ± | 108.84 | 1.44 |
| K139R | 21.23 | ± | 0.32 | 0.53 | 94.03 | ± | 11.04 | 1.40 | 23.27 | ± | 0.51 | 1.47 | 711.00 | ± | 121.24 | 1.38 |
| C141Y | 31.90 | ± | 3.10 | 0.80 | 101.67 | ± | 2.08 | 1.51 | 23.03 | ± | 4.88 | 1.45 | 652.00 | ± | 34.87 | 1.26 |
| P142S | 19.60 | ± | 1.51 | 0.49 | 89.63 | ± | 3.27 | 1.34 | 20.70 | ± | 1.40 | 1.30 | 632.67 | ± | 57.55 | 1.22 |
| V143M | 39.00 | ± | 3.47 | 0.98 | 73.17 | ± | 3.40 | 1.09 | 14.40 | ± | 1.35 | 0.91 | 678.33 | ± | 19.50 | 1.31 |
| Q144* | 35.40 | ± | 2.91 | 0.89 | 98.83 | ± | 4.65 | 1.47 | 12.17 | ± | 0.42 | 0.77 | 674.33 | ± | 10.60 | 1.31 |
| W146* | 20.97 | ± | 0.46 | 0.52 | 74.67 | ± | 3.70 | 1.11 | 20.93 | ± | 2.10 | 1.32 | 695.67 | ± | 25.77 | 1.35 |
| V147I | 20.40 | ± | 0.70 | 0.51 | 69.60 | ± | 2.81 | 1.04 | 21.07 | ± | 0.29 | 1.33 | 653.00 | ± | 3.00 | 1.26 |
| D148G | 43.27 | ± | 0.06 | 1.08 | 88.97 | ± | 1.76 | 1.33 | 16.10 | ± | 0.26 | 1.01 | 688.67 | ± | 53.15 | 1.33 |
| S149 | 22.77 | ± | 1.16 | 0.57 | 71.07 | ± | 7.71 | 1.06 | 17.67 | ± | 0.50 | 1.11 | 689.67 | ± | 8.96 | 1.33 |
| P151S | 19.77 | ± | 1.25 | 0.49 | 169.33 | ± | 10.69 | 2.52 | 14.67 | ± | 2.12 | 0.92 | 674.00 | ± | 8.72 | 1.30 |
| P151L | 20.80 | ± | 0.85 | 0.52 | 248.33 | ± | 4.51 | 3.70 | 12.83 | ± | 2.30 | 0.81 | 881.33 | ± | 27.02 | 1.71 |
| P152 | 42.67 | ± | 0.32 | 1.07 | 259.67 | ± | 3.51 | 3.87 | 20.37 | ± | 2.72 | 1.28 | 587.33 | ± | 11.59 | 1.14 |
| P153S | 66.90 | ± | 0.75 | 1.67 | 96.17 | ± | 2.47 | 1.43 | 15.67 | ± | 0.99 | 0.99 | 701.00 | ± | 16.09 | 1.36 |
| T155A | 19.40 | ± | 0.66 | 0.49 | 56.30 | ± | 2.23 | 0.84 | 14.60 | ± | 0.17 | 0.92 | 591.67 | ± | 49.80 | 1.15 |
| T155I | 36.57 | ± | 2.39 | 0.91 | 77.83 | ± | 1.65 | 1.16 | 28.17 | ± | 0.38 | 1.78 | 792.33 | ± | 12.70 | 1.53 |
| R156H | 49.53 | ± | 0.55 | 1.24 | 57.30 | ± | 0.30 | 0.85 | 13.37 | ± | 1.37 | 0.84 | 476.67 | ± | 135.71 | 0.92 |
| R156C | 53.27 | ± | 0.06 | 1.33 | 123.67 | ± | 1.53 | 1.84 | 17.77 | ± | 0.15 | 1.12 | 653.67 | ± | 37.82 | 1.27 |
| V157I | 30.03 | ± | 0.45 | 0.75 | 78.47 | ± | 2.48 | 1.17 | 19.27 | ± | 0.87 | 1.21 | 663.33 | ± | 11.68 | 1.28 |
| R158L | 51.90 | ± | 2.16 | 1.30 | 76.37 | ± | 8.37 | 1.14 | 14.10 | ± | 2.15 | 0.89 | 708.33 | ± | 35.22 | 1.37 |
| R158H | 55.37 | ± | 4.04 | 1.38 | 55.93 | ± | 4.24 | 0.83 | 15.43 | ± | 1.44 | 0.97 | 617.67 | ± | 11.59 | 1.20 |
| M160I | 57.50 | ± | 1.40 | 1.44 | 103.00 | ± | 1.00 | 1.53 | 9.37 | ± | 0.69 | 0.59 | 406.00 | ± | 9.85 | 0.79 |
| A161V | 53.83 | ± | 6.38 | 1.35 | 63.87 | ± | 0.61 | 0.95 | 23.07 | ± | 0.21 | 1.45 | 583.00 | ± | 12.53 | 1.13 |
| A161T | 51.80 | ± | 2.21 | 1.30 | 117.33 | ± | 3.79 | 1.75 | 14.47 | ± | 1.94 | 0.91 | 852.67 | ± | 95.77 | 1.65 |
| A161P | 58.13 | ± | 2.72 | 1.45 | 72.20 | ± | 7.79 | 1.08 | 15.10 | ± | 0.66 | 0.95 | 465.33 | ± | 19.22 | 0.90 |
| Q167R | 52.87 | ± | 1.42 | 1.32 | 47.23 | ± | 0.59 | 0.70 | 12.33 | ± | 0.06 | 0.78 | 665.00 | ± | 7.94 | 1.29 |
| H168R | 56.23 | ± | 0.25 | 1.41 | 49.13 | ± | 1.68 | 0.73 | 14.53 | ± | 0.45 | 0.92 | 358.00 | ± | 7.94 | 0.69 |
| H168Y | 17.60 | ± | 1.15 | 0.44 | 88.33 | ± | 1.40 | 1.32 | 23.07 | ± | 1.46 | 1.45 | 669.00 | ± | 37.40 | 1.29 |
| V172A | 29.97 | ± | 1.86 | 0.75 | 103.30 | ± | 7.61 | 1.54 | 12.77 | ± | 2.37 | 0.80 | 611.00 | ± | 130.43 | 1.18 |
| R174S | 40.07 | ± | 0.06 | 1.00 | 103.20 | ± | 6.79 | 1.54 | 19.67 | ± | 3.74 | 1.24 | 747.67 | ± | 101.01 | 1.45 |
| C176Y | 61.73 | ± | 4.54 | 1.54 | 142.33 | ± | 25.72 | 2.12 | 13.70 | ± | 2.34 | 0.86 | 763.67 | ± | 52.84 | 1.48 |
| P177T | 13.90 | ± | 0.61 | 0.35 | 58.70 | ± | 2.09 | 0.87 | 23.03 | ± | 1.96 | 1.45 | 567.00 | ± | 45.13 | 1.10 |
| H178ds | 35.07 | ± | 0.32 | 0.88 | 76.80 | ± | 3.40 | 1.14 | 21.97 | ± | 0.90 | 1.38 | 849.67 | ± | 88.01 | 1.64 |
| H178R | 48.00 | ± | 5.27 | 1.20 | 128.67 | ± | 13.32 | 1.92 | 15.17 | ± | 2.15 | 0.96 | 593.67 | ± | 79.05 | 1.15 |
| S183L | 44.50 | ± | 2.17 | 1.11 | 108.00 | ± | 6.93 | 1.61 | 14.63 | ± | 1.96 | 0.92 | 751.67 | ± | 63.57 | 1.45 |
| D186N | 69.63 | ± | 2.66 | 1.74 | 91.67 | ± | 0.58 | 1.37 | 34.93 | ± | 6.02 | 2.20 | 960.33 | ± | 26.41 | 1.86 |
| A189T | 70.83 | ± | 2.45 | 1.77 | 96.43 | ± | 3.35 | 1.44 | 13.17 | ± | 2.82 | 0.83 | 953.33 | ± | 38.81 | 1.85 |
| Q192L | 45.97 | ± | 3.41 | 1.15 | 64.27 | ± | 21.95 | 0.96 | 10.30 | ± | 1.05 | 0.65 | 678.67 | ± | 13.28 | 1.31 |
| H193Y | 40.17 | ± | 1.16 | 1.00 | 154.33 | ± | 16.86 | 2.30 | 18.77 | ± | 2.25 | 1.18 | 521.67 | ± | 44.97 | 1.01 |
| L194F | 73.30 | ± | 1.78 | 1.83 | 109.90 | ± | 17.48 | 1.64 | 12.97 | ± | 4.33 | 0.82 | 536.33 | ± | 85.05 | 1.04 |
| R196* | 61.30 | ± | 2.36 | 1.53 | 81.53 | ± | 1.71 | 1.21 | 16.97 | ± | 1.33 | 1.07 | 690.00 | ± | 64.28 | 1.34 |
| R202C | 48.83 | ± | 5.82 | 1.22 | 88.03 | ± | 2.73 | 1.31 | 18.07 | ± | 1.04 | 1.14 | 597.33 | ± | 83.07 | 1.16 |
| R202S | 37.07 | ± | 1.10 | 0.93 | 138.67 | ± | 8.50 | 2.07 | 14.60 | ± | 0.30 | 0.92 | 276.33 | ± | 15.18 | 0.53 |
| T211I | 47.83 | ± | 3.95 | 1.20 | 90.17 | ± | 1.21 | 1.34 | 13.77 | ± | 0.64 | 0.87 | 618.67 | ± | 31.53 | 1.20 |
| R213* | 73.87 | ± | 20.45 | 1.85 | 149.00 | ± | 6.93 | 2.22 | 36.17 | ± | 0.874 | 2.28 | 966.33 | ± | 12.70 | 1.87 |
| V217M | 74.00 | ± | 27.46 | 1.85 | 95.50 | ± | 1.73 | 1.42 | 24.80 | ± | 0.17 | 1.56 | 386.33 | ± | 5.51 | 0.75 |
| E221* | 30.57 | ± | 0.93 | 0.76 | 77.13 | ± | 1.79 | 1.15 | 10.05 | ± | 0.69 | 0.63 | 378.33 | ± | 19.86 | 0.73 |
| P223H | 39.77 | ± | 0.46 | 0.99 | 75.87 | ± | 2.15 | 1.13 | 18.67 | ± | 0.71 | 1.18 | 584.67 | ± | 46.19 | 1.13 |
| G226D | 52.60 | ± | 7.69 | 1.32 | 84.00 | ± | 5.48 | 1.25 | 16.70 | ± | 0.95 | 1.05 | 480.33 | ± | 102.26 | 0.93 |
| S227P | 81.53 | ± | 3.76 | 2.04 | 94.17 | ± | 1.26 | 1.40 | 16.50 | ± | 0.26 | 1.04 | 820.00 | ± | 148.84 | 1.59 |
| D228 | 60.53 | ± | 5.87 | 1.51 | 92.63 | ± | 1.76 | 1.38 | 29.43 | ± | 1.37 | 1.86 | 507.67 | ± | 41.40 | 0.98 |
| C229 | 67.10 | ± | 1.61 | 1.68 | 83.43 | ± | 1.43 | 1.24 | 18.70 | ± | 3.73 | 1.18 | 539.00 | ± | 84.12 | 1.04 |
| T231I | 58.43 | ± | 3.01 | 1.46 | 81.13 | ± | 7.67 | 1.21 | 16.20 | ± | 1.45 | 1.02 | 409.33 | ± | 135.00 | 0.79 |
| I232M | 78.70 | ± | 1.48 | 1.97 | 87.67 | ± | 1.37 | 1.31 | 8.73 | ± | 0.29 | 0.55 | 270.00 | ± | 61.51 | 0.52 |
| H233R | 45.23 | ± | 1.86 | 1.13 | 59.93 | ± | 3.05 | 0.89 | 15.73 | ± | 1.56 | 0.99 | 657.00 | ± | 33.87 | 1.27 |
| Y234C | 27.87 | ± | 2.87 | 0.70 | 151.67 | ± | 10.07 | 2.26 | 8.53 | ± | 0.90 | 0.54 | 264.67 | ± | 42.72 | 0.51 |
| N235D | 26.93 | ± | 1.56 | 0.67 | 151.67 | ± | 14.01 | 2.26 | 8.82 | ± | 1.03 | 0.56 | 246.00 | ± | 140.27 | 0.48 |
| N235I | 36.37 | ± | 2.32 | 0.91 | 93.27 | ± | 2.63 | 1.39 | 12.80 | ± | 0.36 | 0.81 | 592.00 | ± | 90.42 | 1.15 |
| N235S | 55.67 | ± | 2.96 | 1.39 | 77.40 | ± | 2.40 | 1.15 | 24.33 | ± | 1.17 | 1.53 | 654.67 | ± | 52.52 | 1.27 |
| M237T | 59.73 | ± | 3.63 | 1.49 | 78.60 | ± | 2.14 | 1.17 | 13.23 | ± | 3.82 | 0.83 | 309.00 | ± | 91.07 | 0.60 |
| C238Y | 35.50 | ± | 0.40 | 0.89 | 112.00 | ± | 1.00 | 1.67 | 18.70 | ± | 0.66 | 1.18 | 637.00 | ± | 15.59 | 1.23 |
| N239S | 80.00 | ± | 4.23 | 2.00 | 147.67 | ± | 7.57 | 2.20 | 31.10 | ± | 0.85 | 1.96 | 981.00 | ± | | 1.90 |
| M234V | 48.80 | ± | 5.84 | 1.22 | 77.53 | ± | 2.44 | 1.15 | 13.37 | ± | 2.22 | 0.84 | 485.00 | ± | 113.96 | 0.94 |
| G244D | 48.87 | ± | 3.10 | 1.22 | 142.00 | ± | 1.73 | 2.12 | 18.30 | ± | 3.44 | 1.15 | 705.67 | ± | 2.52 | 1.37 |
| G245D | 39.23 | ± | 0.71 | 0.98 | 93.07 | ± | 5.90 | 1.39 | 19.03 | ± | 0.31 | 1.20 | 461.33 | ± | 40.55 | 0.89 |
| M246V | 37.00 | ± | 2.21 | 0.93 | 96.40 | ± | 24.17 | 1.44 | 13.33 | ± | 0.57 | 0.84 | 566.67 | ± | 25.70 | 1.10 |
| R248Q | 94.47 | ± | 3.86 | 2.36 | 294.33 | ± | 4.73 | 4.38 | 81.03 | ± | 4.21 | 5.11 | >1000 | ± | |  |
| R248W | 83.80 | ± | 1.47 | 2.10 | 146.33 | ± | 3.21 | 2.18 | 59.37 | ± | 10.24 | 3.74 | >1000 | ± | |  |
| R249G | 29.80 | ± | 3.08 | 0.75 | 31.13 | ± | 1.03 | 0.46 | 7.05 | ± | 0.36 | 0.44 | 270.67 | ± | 63.52 | 0.52 |
| P250L | 72.00 | ± | 0.61 | 1.80 | 215.67 | ± | 38.84 | 3.21 | 7.97 | ± | 0.94 | 0.50 | 619.00 | ± | 7.81 | 1.20 |
| I251M | 29.87 | ± | 1.91 | 0.75 | 159.33 | ± | 29.77 | 2.37 | 15.33 | ± | 1.68 | 0.97 | 542.33 | ± | 84.11 | 1.05 |
| I255T | 41.73 | ± | 0.06 | 1.04 | 132.00 | ± | 11.53 | 1.97 | 15.17 | ± | 0.61 | 0.96 | 719.67 | ± | 40.02 | 1.39 |
| T256A | 76.97 | ± | 5.76 | 1.92 | 95.97 | ± | 7.87 | 1.43 | 29.50 | ± | 1.04 | 1.86 | 613.00 | ± | 47.63 | 1.19 |
| L257P | 35.07 | ± | 0.46 | 0.88 | 85.47 | ± | 1.53 | 1.27 | 21.47 | ± | 1.50 | 1.35 | 605.00 | ± | 82.16 | 1.17 |
| L257E | 26.10 | ± | 2.01 | 0.65 | 166.33 | ± | 8.50 | 2.48 | 19.93 | ± | 1.50 | 1.26 | 516.00 | ± | 26.66 | 1.00 |
| L257M | 56.83 | ± | 5.33 | 1.42 | 82.33 | ± | 0.85 | 1.23 | 14.90 | ± | 1.65 | 0.94 | 557.67 | ± | 18.90 | 1.08 |
| D259G | 52.57 | ± | 1.60 | 1.31 | 60.47 | ± | 2.70 | 0.90 | 21.03 | ± | 0.60 | 1.33 | 661.33 | ± | 31.63 | 1.28 |
| G266R | 53.00 | ± | 1.87 | 1.33 | 109.33 | ± | 9.29 | 1.63 | 11.30 | ± | 0.80 | 0.71 | 700.00 | ± | 21.17 | 1.35 |
| R267Q | 58.43 | ± | 3.01 | 1.46 | 56.57 | ± | 2.31 | 0.84 | 19.00 | ± | 0.36 | 1.20 | 615.33 | ± | 15.01 | 1.19 |
| S269G | 32.37 | ± | 0.61 | 0.81 | 112.67 | ± | 4.04 | 1.68 | 13.20 | ± | 0.20 | 0.83 | 725.33 | ± | 8.62 | 1.40 |
| S269N | 34.33 | ± | 1.00 | 0.86 | 81.67 | ± | 5.88 | 1.22 | 18.00 | ± | 1.41 | 1.13 | 280.33 | ± | 65.74 | 0.54 |
| F270L | 48.93 | ± | 0.31 | 1.22 | 65.10 | ± | 1.55 | 0.97 | 13.87 | ± | 1.07 | 0.87 | 567.67 | ± | 2.31 | 1.10 |
| E271K | 41.27 | ± | 1.31 | 1.03 | 96.10 | ± | 7.05 | 1.43 | 13.70 | ± | 1.21 | 0.86 | 575.00 | ± | 36.43 | 1.11 |
| H273C | 35.23 | ± | 1.53 | 0.88 | 166.67 | ± | 2.08 | 2.48 | 21.40 | ± | 0.75 | 1.35 | 461.00 | ± | 3.61 | 0.89 |
| V274I | 82.00 | ± | 0.62 | 2.05 | 151.33 | ± | 17.79 | 2.25 | 19.80 | ± | 0.44 | 1.25 | 607.33 | ± | 20.31 | 1.18 |
| A276V | 74.80 | ± | 7.97 | 1.87 | 81.77 | ± | 3.88 | 1.22 | 16.70 | ± | 2.14 | 1.05 | 586.67 | ± | 55.51 | 1.14 |
| R280T | 39.17 | ± | 0.49 | 0.98 | 116.00 | ± | 4.58 | 1.73 | 15.27 | ± | 1.27 | 0.96 | 627.33 | ± | 18.04 | 1.21 |
| R280L | 22.43 | ± | 1.81 | 0.56 | 153.33 | ± | 2.89 | 2.28 | 12.43 | ± | 0.97 | 0.78 | 797.67 | ± | 13.01 | 1.54 |
| R282Q | 75.67 | ± | 4.91 | 1.89 | 85.43 | ± | 5.11 | 1.27 | 19.13 | ± | 1.10 | 1.21 | 415.67 | ± | 21.36 | 0.80 |
| P295S | 69.70 | ± | 6.94 | 1.74 | 58.13 | ± | 2.31 | 0.87 | 19.73 | ± | 3.10 | 1.24 | 433.33 | ± | 22.81 | 0.84 |
| H297R | 31.30 | ± | 1.06 | 0.78 | 97.40 | ± | 0.87 | 1.45 | 9.59 | ± | 1.57 | 0.60 | 712.33 | ± | 8.33 | 1.38 |
| P300S | 49.50 | ± | 1.91 | 1.24 | 121.33 | ± | 17.47 | 1.81 | 8.34 | ± | 0.58 | 0.53 | 202.67 | ± | 66.98 | 0.39 |
| P305R | 22.60 | ± | 1.68 | 0.57 | 73.63 | ± | 1.91 | 1.10 | 12.80 | ± | 1.80 | 0.81 | 757.00 | ± | 20.07 | 1.47 |
| R306* | 24.00 | ± | 0.95 | 0.60 | 79.90 | ± | 1.93 | 1.19 | 15.90 | ± | 0.46 | 1.00 | 815.67 | ± | 48.76 | 1.58 |
| P316S | 25.40 | ± | 2.07 | 0.64 | 70.43 | ± | 2.32 | 1.05 | 9.84 | ± | 0.16 | 0.62 | 648.67 | ± | 137.35 | 1.26 |
| Q317H | 67.27 | ± | 4.02 | 1.68 | 83.40 | ± | 1.25 | 1.24 | 13.37 | ± | 0.81 | 0.84 | 948.33 | ± | 44.88 | 1.84 |
| P318S | 14.37 | ± | 2.10 | 0.36 | 71.43 | ± | 4.48 | 1.06 | 14.37 | ± | 1.06 | 0.91 | 681.67 | ± | 60.17 | 1.32 |
| K321E | 20.70 | ± | 2.84 | 0.52 | 71.97 | ± | 3.41 | 1.07 | 14.30 | ± | 0.61 | 0.90 | 698.00 | ± | 40.04 | 1.35 |
| D324N | 22.47 | ± | 2.63 | 0.56 | 77.60 | ± | 1.04 | 1.16 | 14.53 | ± | 3.96 | 0.92 | 719.33 | ± | 33.25 | 1.39 |
| R333H | 20.97 | ± | 2.47 | 0.52 | 78.17 | ± | 2.62 | 1.16 | 15.07 | ± | 2.84 | 0.95 | 786.00 | ± | 1.73 | 1.52 |
| R337S | 21.83 | ± | 3.40 | 0.55 | 74.83 | ± | 4.18 | 1.11 | 13.57 | ± | 1.22 | 0.86 | 860.33 | ± | 64.58 | 1.67 |
| M340T | 24.27 | ± | 2.61 | 0.61 | 78.60 | ± | 1.73 | 1.17 | 16.97 | ± | 2.73 | 1.07 | 608.00 | ± | 39.61 | 1.18 |
| R342* | 20.80 | ± | 2.07 | 0.52 | 77.97 | ± | 3.70 | 1.16 | 15.53 | ± | 0.64 | 0.98 | 902.33 | ± | 86.50 | 1.75 |
| R342Q | 84.70 | ± | 3.27 | 2.12 | 158.33 | ± | 3.21 | 2.36 | 17.80 | ± | 2.33 | 1.12 | >1000 | ± | |  |
| N345D | 21.10 | ± | 2.72 | 0.53 | 73.57 | ± | 4.07 | 1.10 | 15.00 | ± | 3.20 | 0.95 | 823.33 | ± | 155.05 | 1.59 |
| G360R | 29.53 | ± | 1.75 | 0.74 | 78.93 | ± | 1.72 | 1.18 | 16.00 | ± | 0.50 | 1.01 | 870.00 | ± | 23.26 | 1.68 |
| S362G | 23.43 | ± | 5.63 | 0.59 | 76.50 | ± | 4.61 | 1.14 | 18.73 | ± | 1.90 | 1.18 | 875.00 | ± | 124.69 | 1.69 |
| H365Y | 30.03 | ± | 2.37 | 0.75 | 79.47 | ± | 1.75 | 1.18 | 14.47 | ± | 2.34 | 0.91 | 958.00 | ± | 41.33 | 1.85 |
| L369fs | 23.43 | ± | 0.23 | 0.59 | 75.77 | ± | 1.79 | 1.13 | 16.40 | ± | 2.19 | 1.03 | 973.00 | ± | 22.11 | 1.88 |
| Q375* | 19.60 | ± | 2.96 | 0.49 | 79.57 | ± | 1.22 | 1.19 | 15.80 | ± | 0.17 | 1.00 | 510.33 | ± | 66.02 | 0.99 |

RF: resistant factor; RF= IC_50_ of WT or mutant / IC_50_ of control; Leflunomide (LEF); Hydroxychloroquine (HCQ); Cyclosporine A (CSA); Tacrolimus (FK506)

## Original western blot for three repeats

**
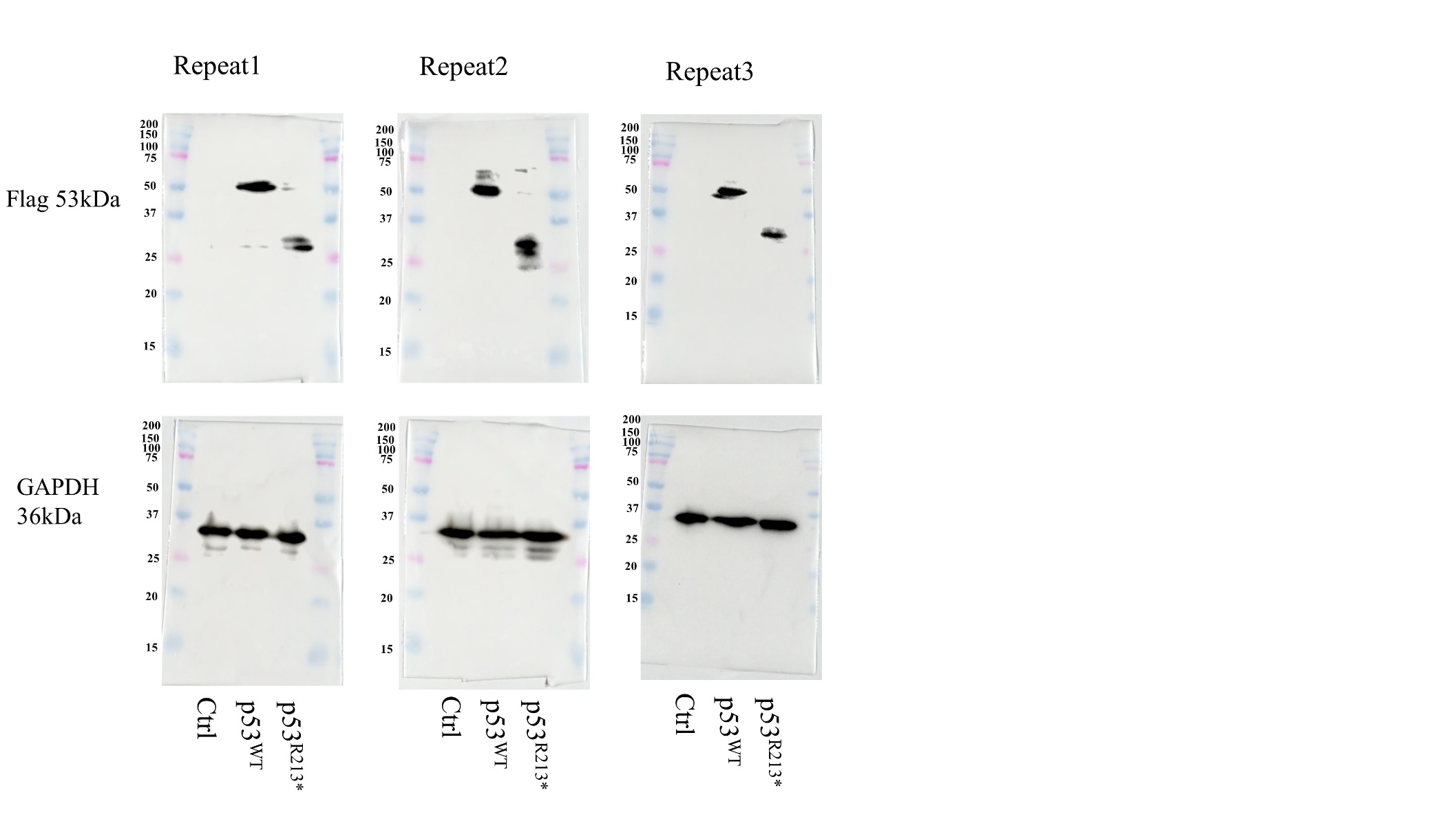

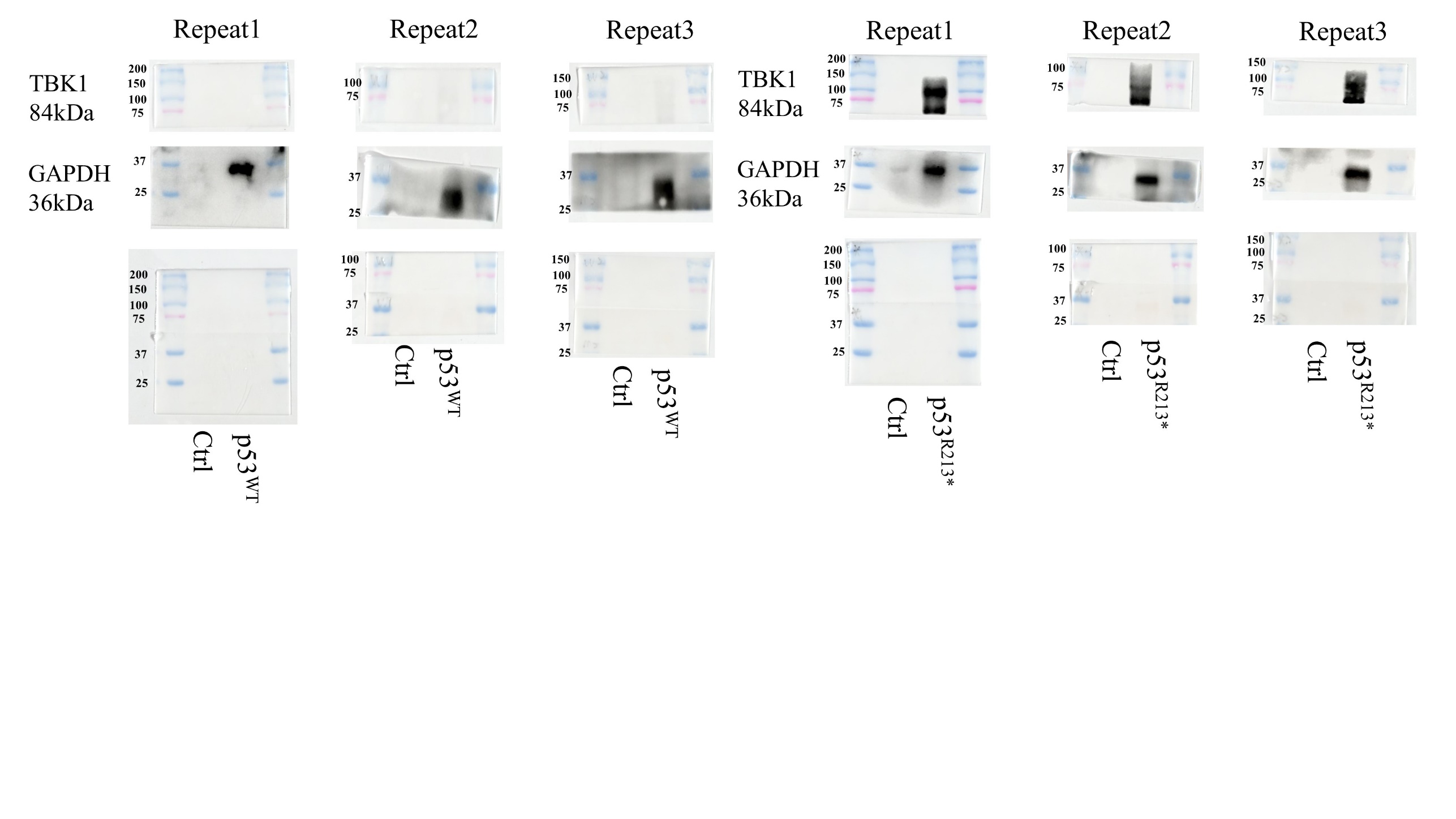

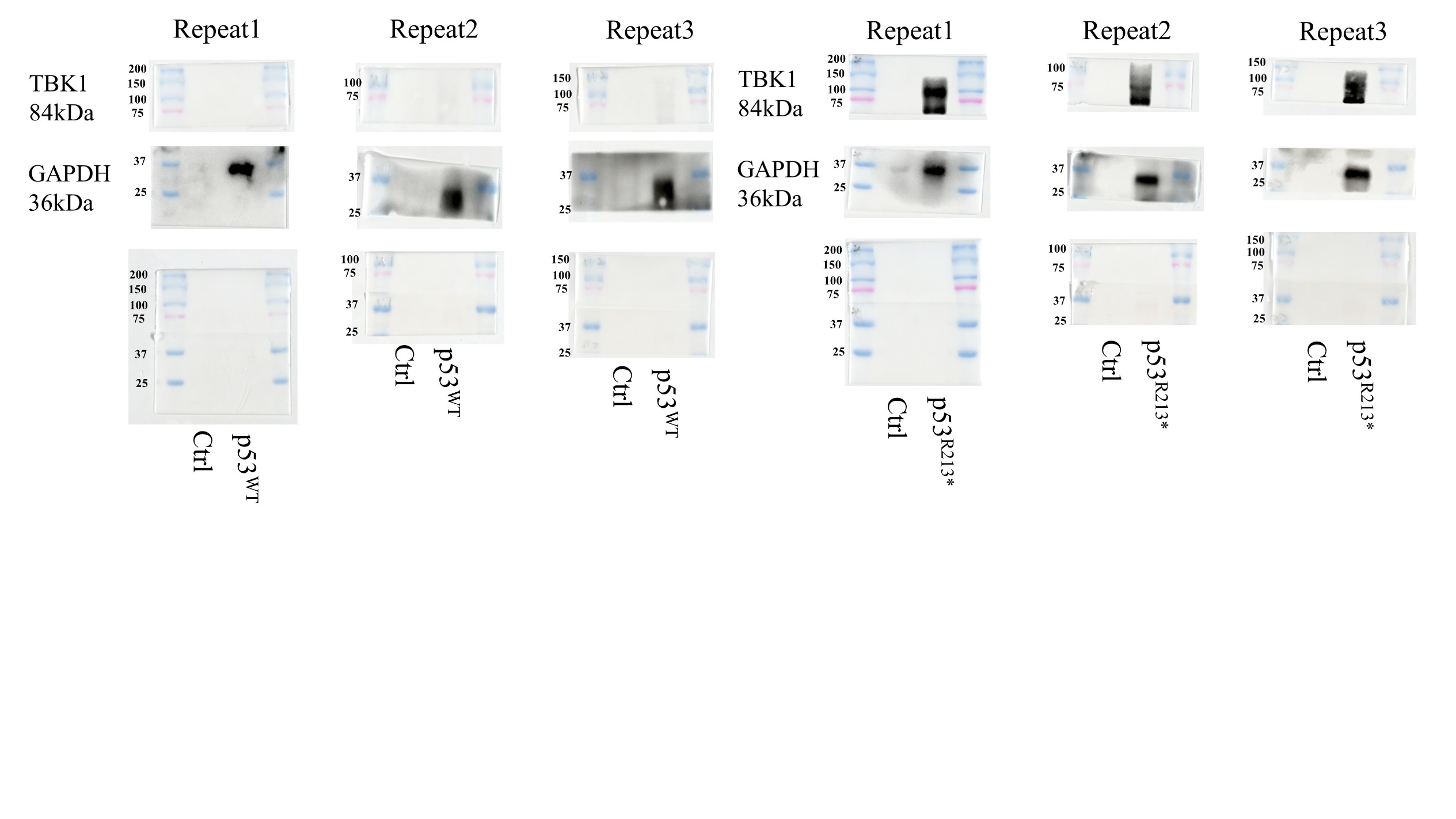

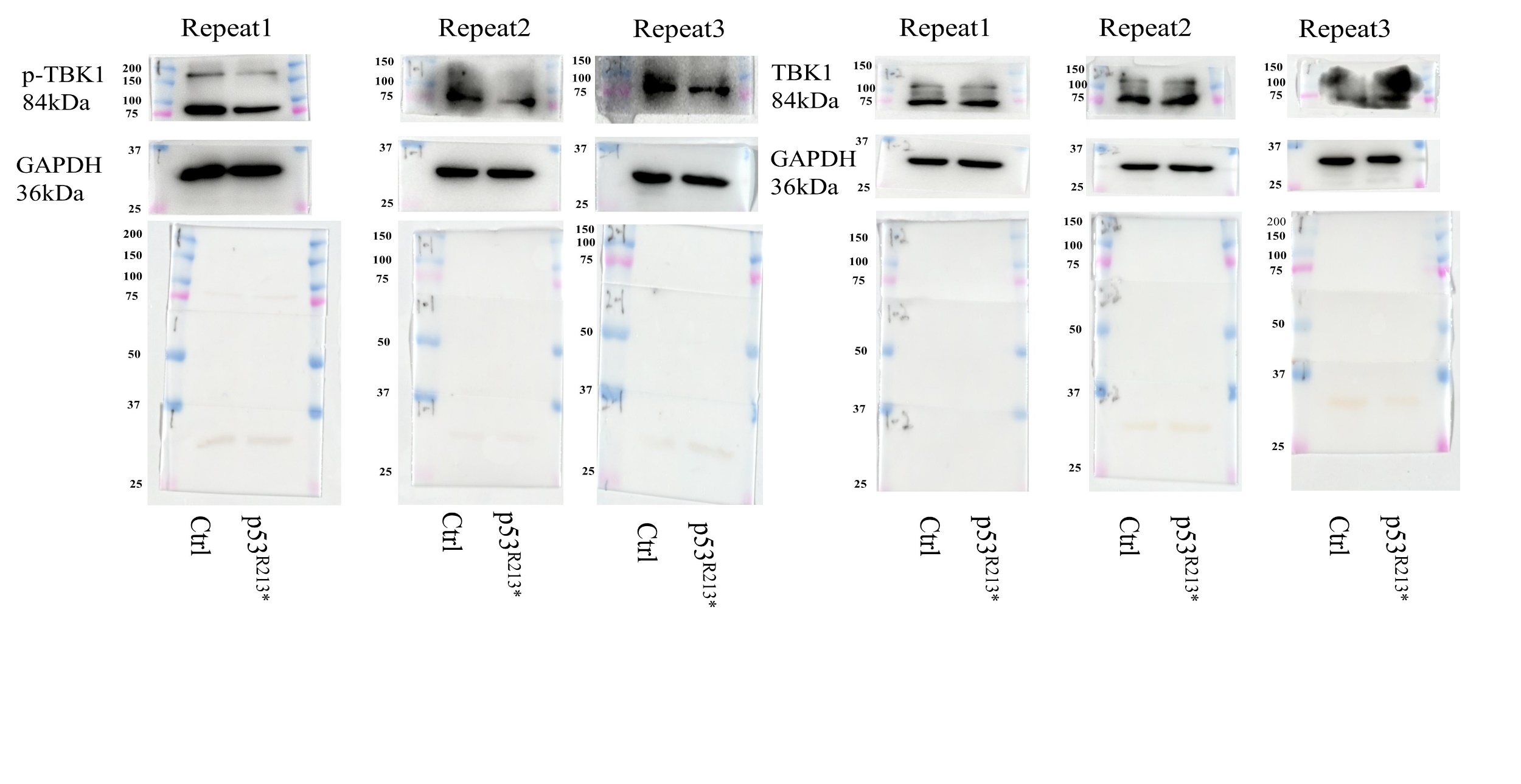

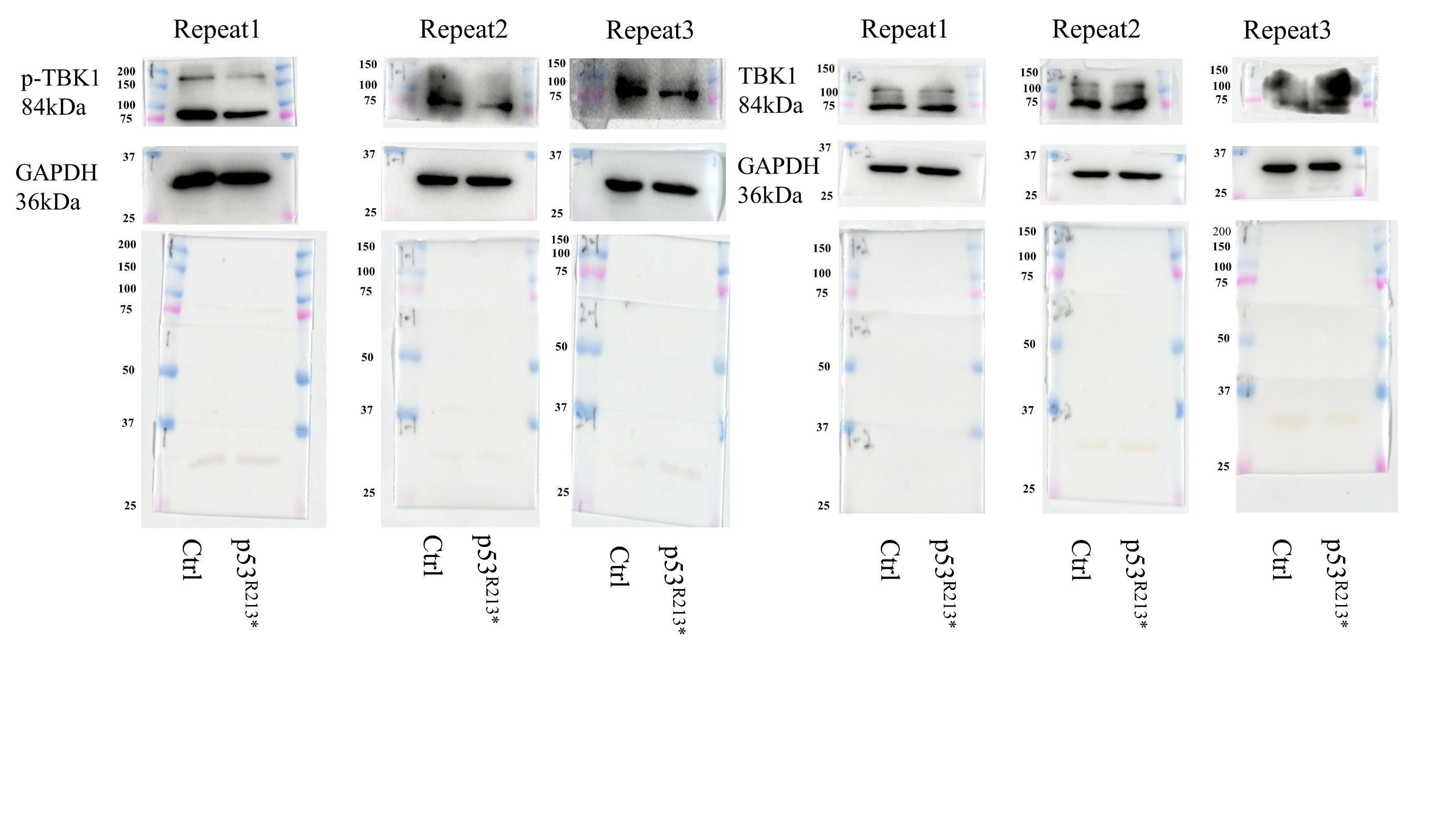

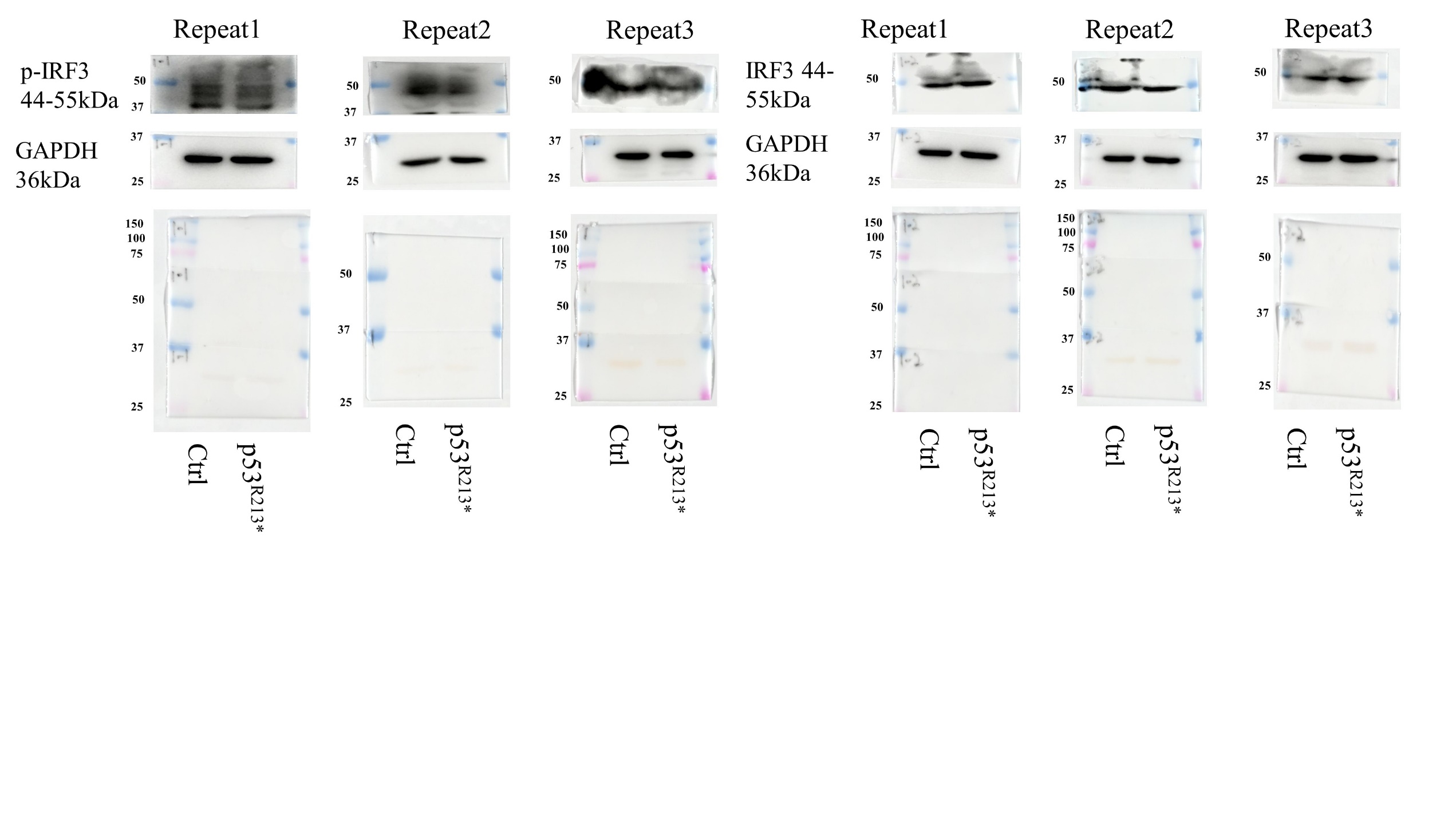

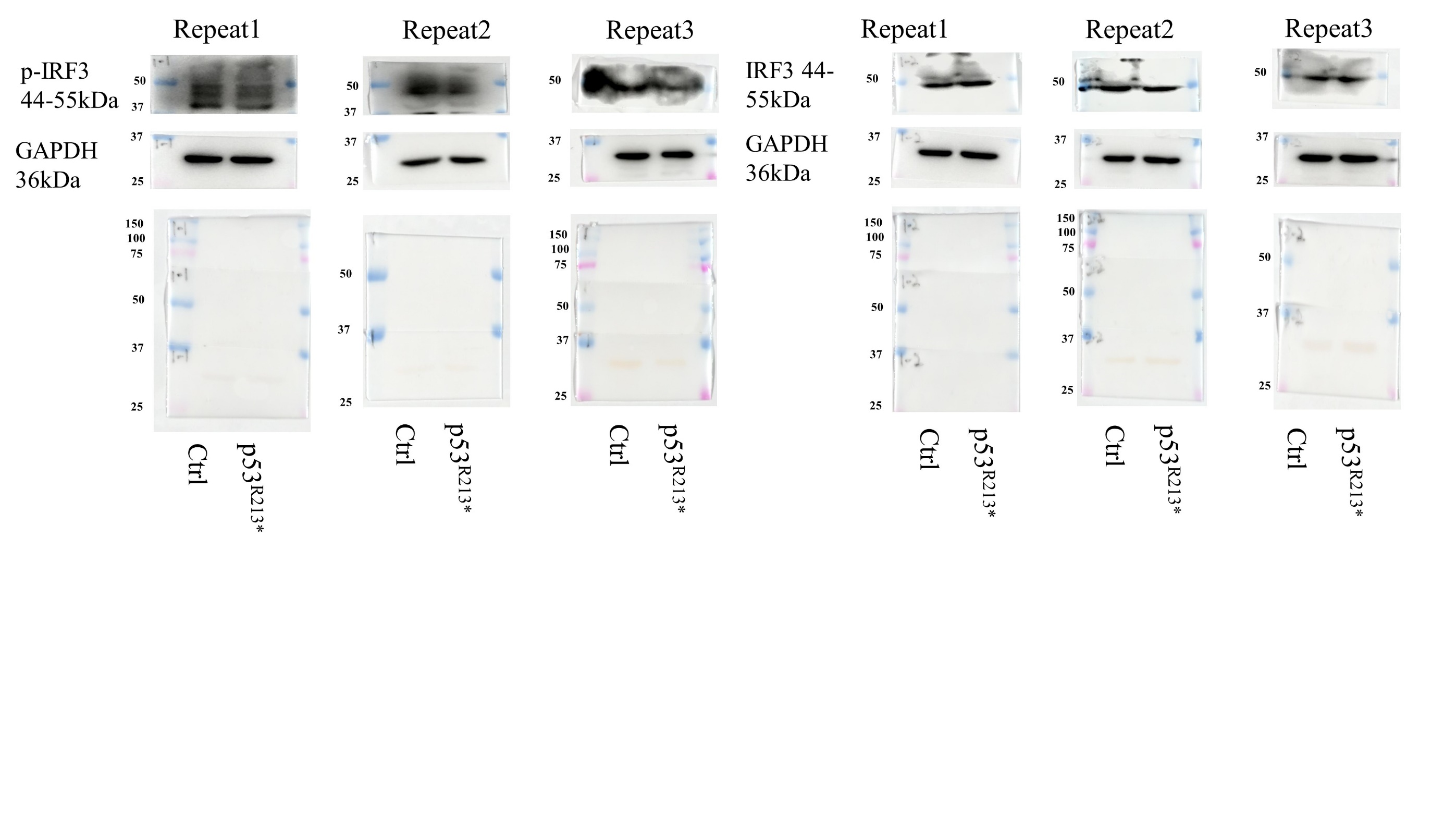
**

**
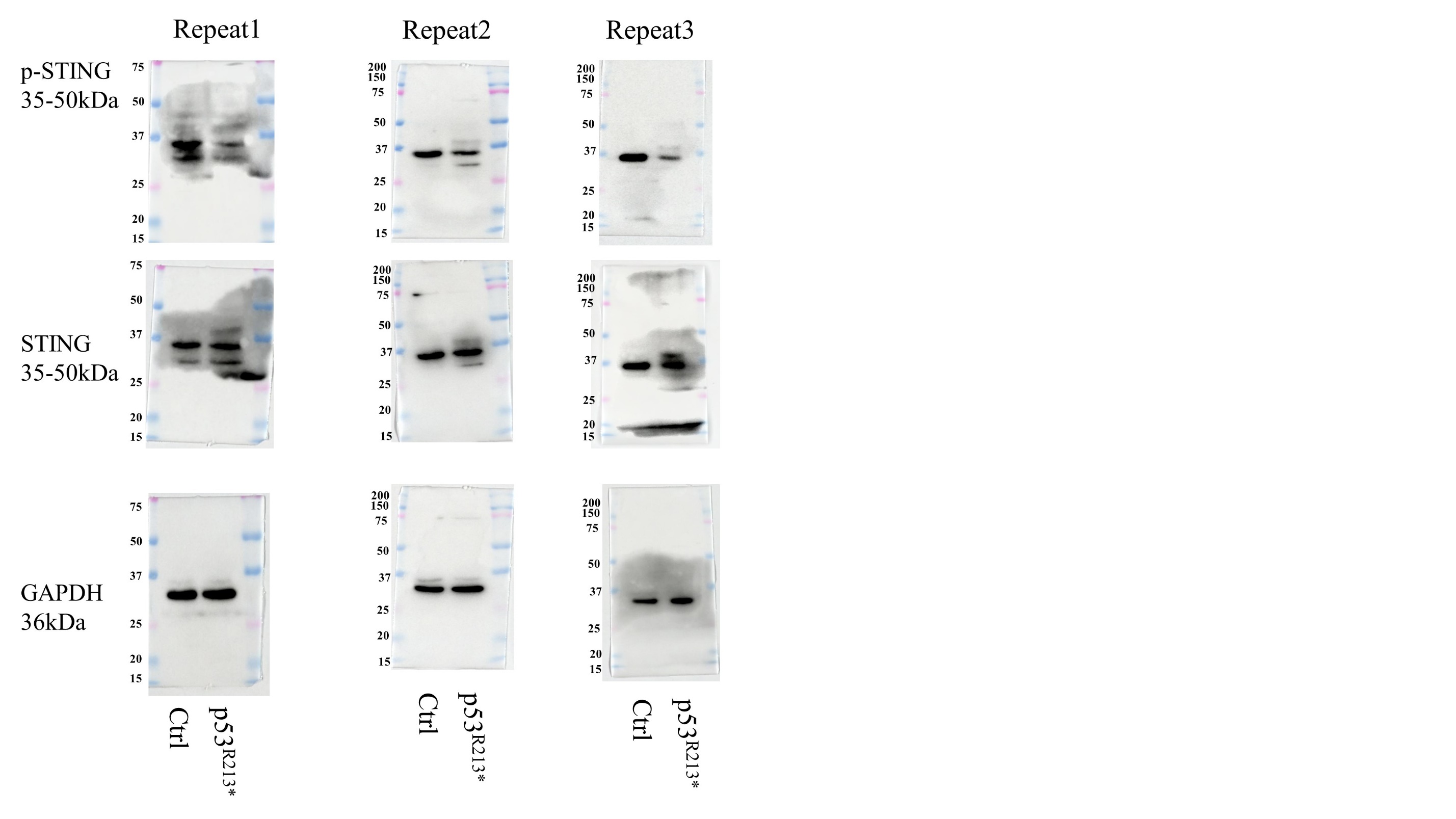

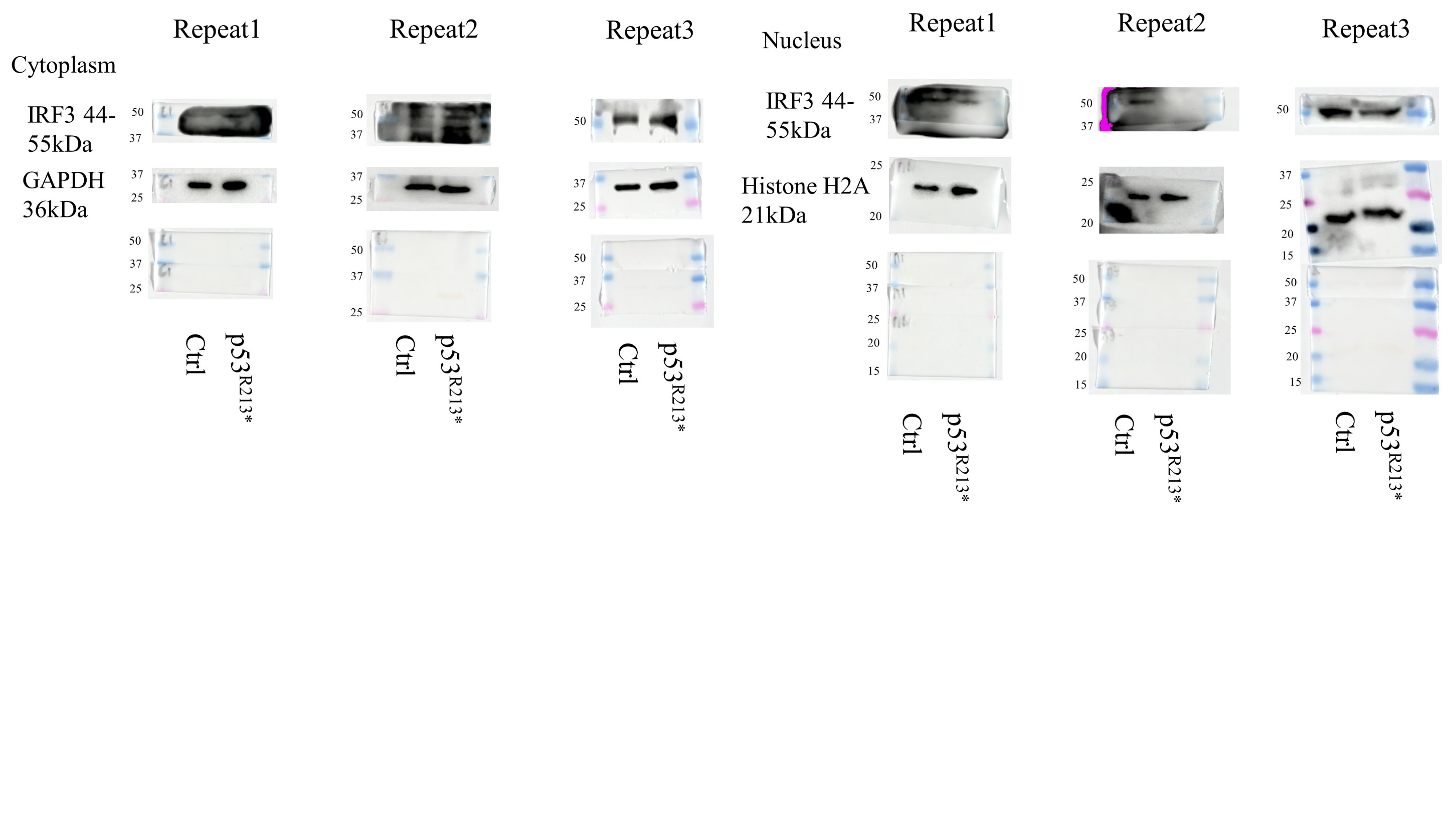

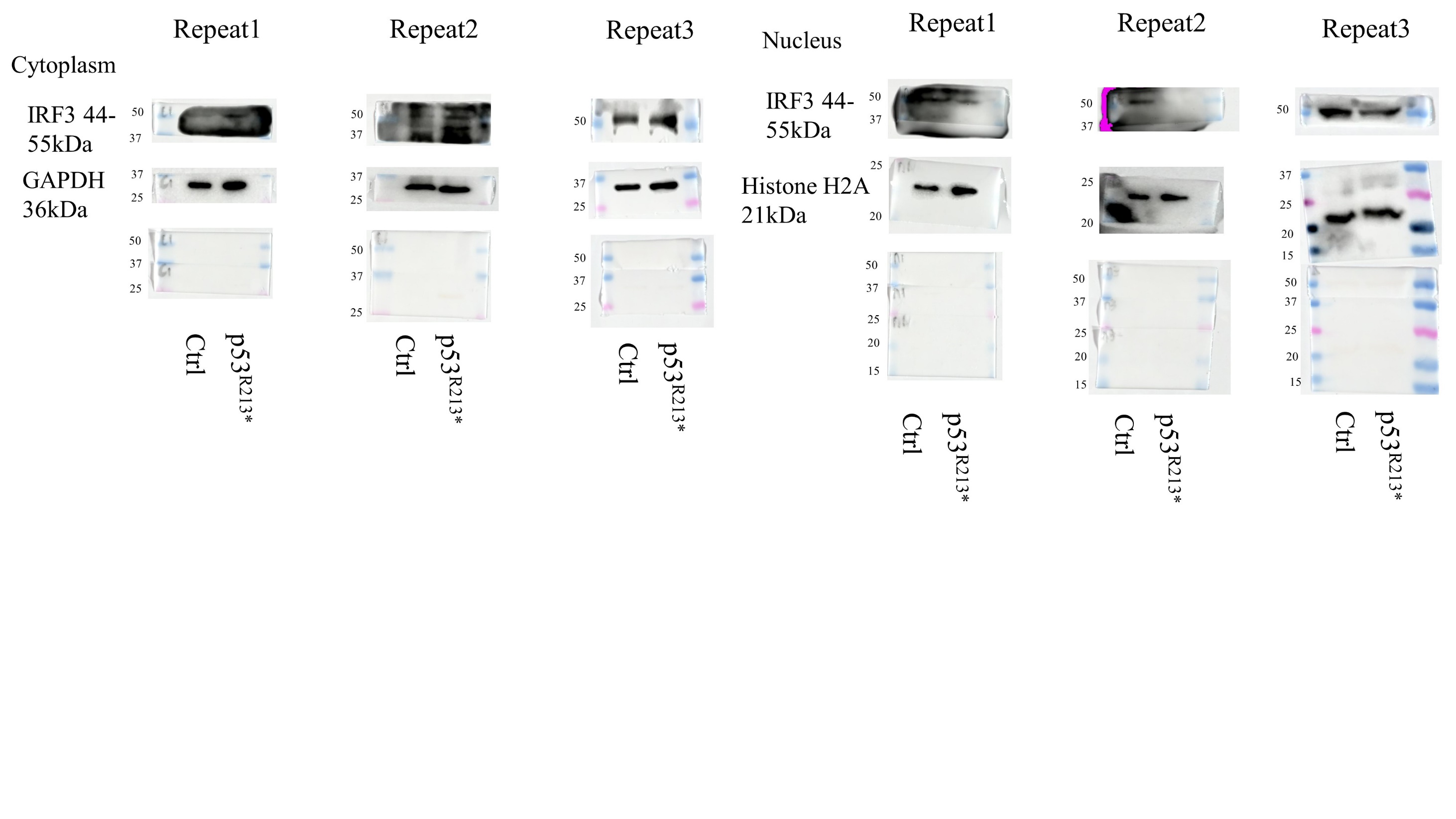
**
